# Distinctive Roles of Two Acinetobactin Isomers in Challenging Host Nutritional Immunity

**DOI:** 10.1128/mBio.02248-21

**Published:** 2021-09-14

**Authors:** Mingi Kim, Do Young Kim, Woon Young Song, So Eun Park, Simone A. Harrison, Walter J. Chazin, Man Hwan Oh, Hak Joong Kim

**Affiliations:** a Department of Chemistry, Korea University, Seoul, Republic of Korea; b Center for ProteoGenomics Research, Korea University, Seoul, Republic of Korea; c Department of Biochemistry, Vanderbilt University, Nashville, Tennessee, USA; d Department of Chemistry, Vanderbilt University, Nashville, Tennessee, USA; e Center for Structural Biology, Vanderbilt University, Nashville, Tennessee, USA; f Department of Microbiology, Dankook University, Cheonan, Republic of Korea; Kansas State University; McMaster University

**Keywords:** *Acinetobacter baumannii*, iron metabolism, nutritional immunity, siderophore, virulence factors

## Abstract

The human pathogen Acinetobacter baumannii produces and utilizes acinetobactin for iron assimilation. Although two isomeric structures of acinetobactin, one featuring an oxazoline (Oxa) and the other with an isoxazolidinone (Isox) at the core, have been identified, their differential roles as virulence factors for successful infection have yet to be established. This study provides direct evidence that Oxa supplies iron more efficiently than Isox, primarily owing to its specific recognition by the cognate outer membrane receptor, BauA. The other components in the acinetobactin uptake machinery appear not to discriminate these isomers. Interestingly, Oxa was found to form a stable iron complex that is resistant to release of the chelated iron upon competition by Isox, despite their comparable apparent affinities to Fe(III). In addition, both Oxa and Isox were found to be competent iron chelators successfully scavenging iron from host metal sequestering proteins responsible for nutritional immunity. These observations collectively led us to propose a new model for acinetobactin-based iron assimilation at infection sites. Namely, Oxa is the principal siderophore mediating the core Fe(III) supply chain for A. baumannii, whereas Isox plays a minor role in the iron delivery and, alternatively, functions as an auxiliary iron collector that channels the iron pool toward Oxa. The unique siderophore utilization mechanism proposed here represents an intriguing strategy for pathogen adaptation under the various nutritional stresses encountered at infection sites.

## INTRODUCTION

Acinetobacter baumannii has emerged as a serious threat to human health, particularly in nosocomial situations (1, 2). The recent drastic increase in multidrug-resistant A. baumannii strains is particularly daunting because it significantly limits the therapeutic options for treating infected patients, who are immunocompromised in many cases and, thus, highly vulnerable to this deadly pathogen. To overcome the drug resistance problem of A. baumannii, the efficacy of various new therapeutic modalities, as alternatives or complements to conventional antibiotics, is being actively tested (3–6).

A strategy based on the development of antivirulence agents is attracting particular attention (6–8). Among the various virulence factors, those with mechanisms associated with the acquisition of essential nutrients, particularly iron, were proposed to be well suited for antivirulence therapy (9–11). During infection, invading bacteria face the restriction of essential nutrients by the host immune defense system; this often involves secretion of proteins capable of forming tight complexes with nutrients, thereby limiting their access by the pathogen. This mechanism is often referred to as “nutritional immunity” (9, 12). To overcome limited iron availability at infection sites, A. baumannii primarily relies on two active iron acquisition systems, the ferrous iron transport system for Fe(II) and siderophore-based mechanisms for Fe(III). Recently, Visca and coworkers demonstrated that the latter, not the former, appears to be essential for virulence based on *in vivo* studies, using insect and mouse models (13).

**FIG 1:**
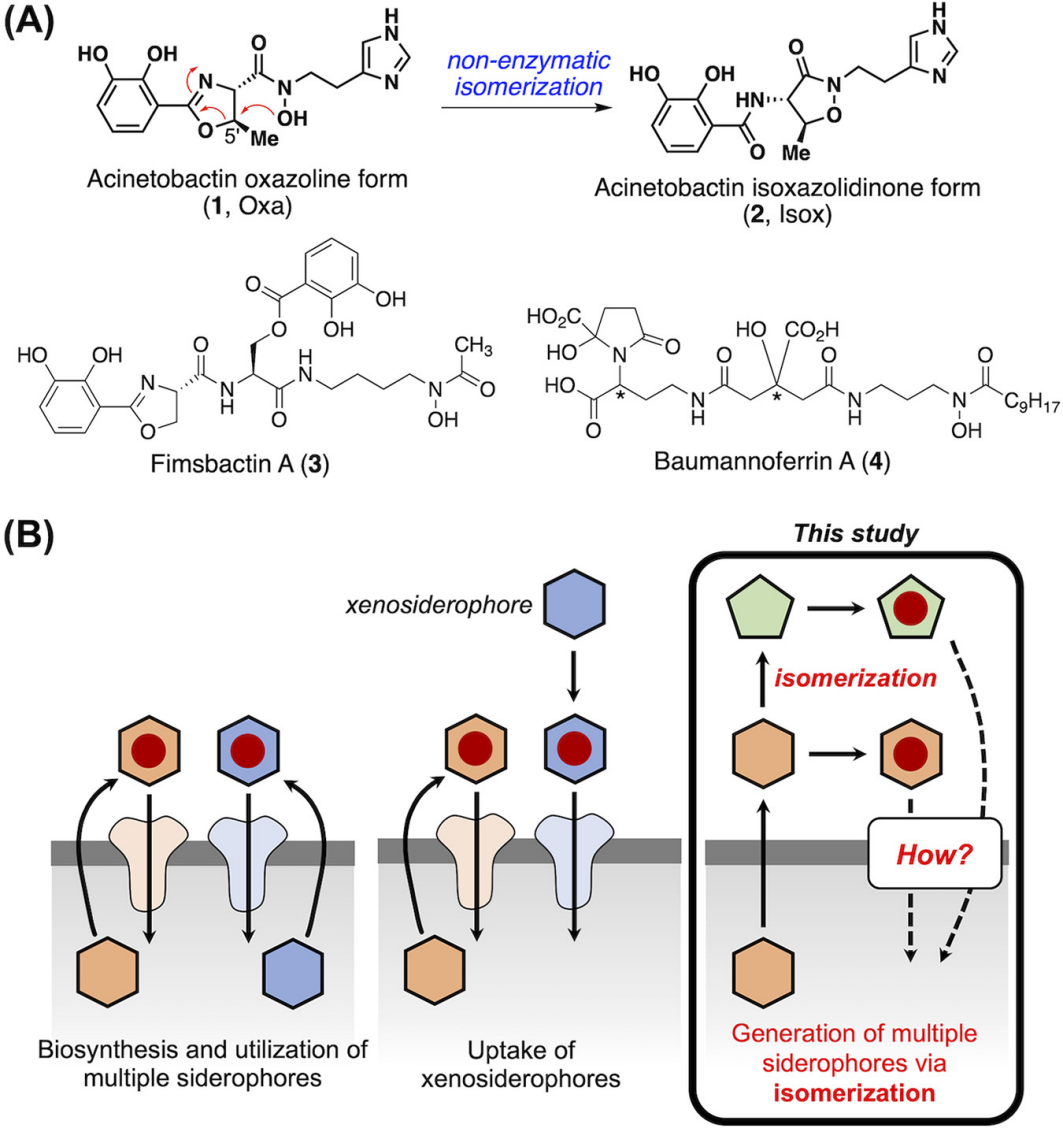
Scope of the current study. (A) The structures of native siderophores produced and utilized by Acinetobacter baumannii. (B) Three different models for multiple siderophore utilization in bacterial iron assimilation. This study is focused on elucidating the mode of acinetobactin utilization, which is unique in that two structurally related siderophores emerge by isomerization from one to the other (the third model).

**FIG 2:**
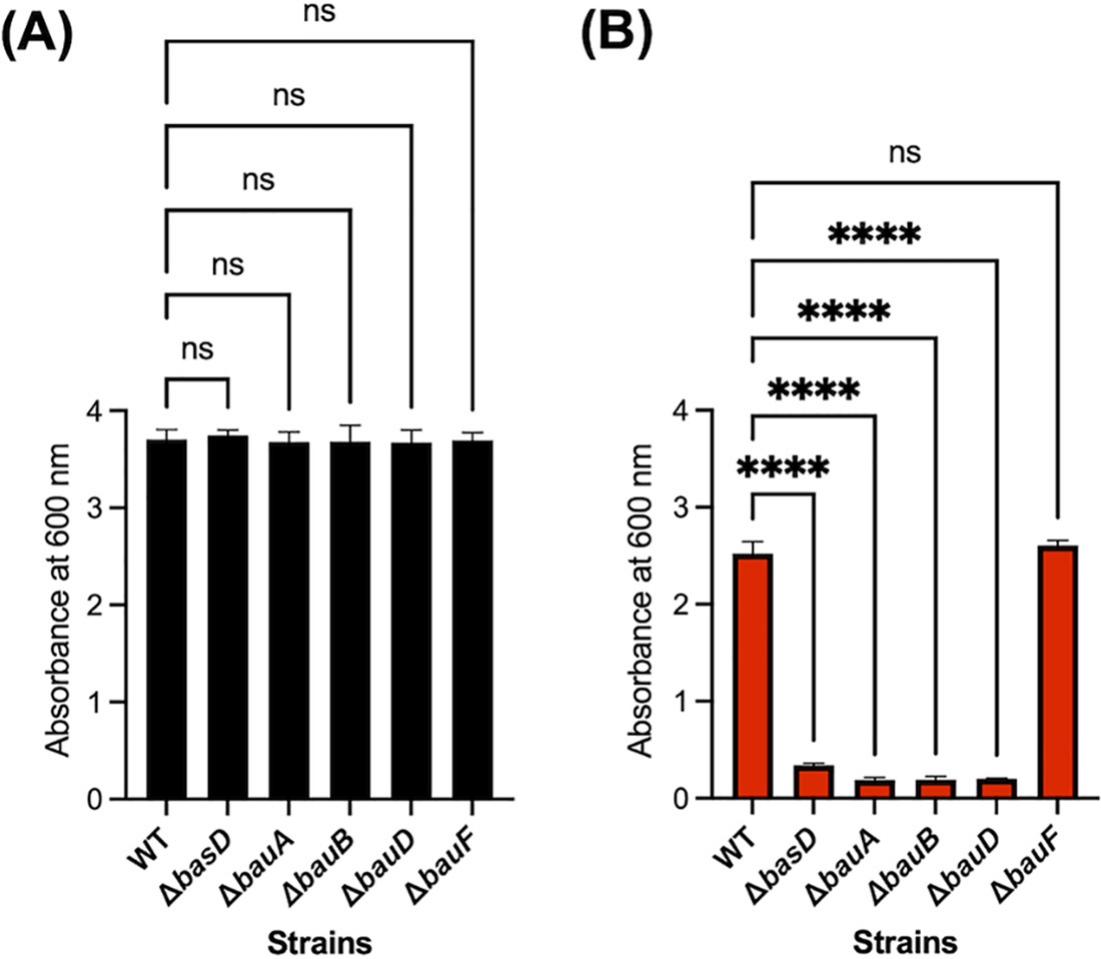
Impact of gene disruption on the growth under iron-deficient conditions. (A and B) Growth of the A. baumannii ATCC 19606 wild type (WT), Δ*basD*, Δ*bauA*, Δ*bauB*, Δ*bauD*, and Δ*bauF* in the LB medium (A) without or (B) with 200 μM DP. The growth of each bacterial strain was measured using a 1-cm cuvette after 24 h of incubation at 37°C. Error bars represent the standard deviations of three independent experiments. Statistical significance was assessed by one-way analysis of variance (ANOVA) tests (ns, not significant; ****, *P < *0.0001).

A. baumannii is known to produce and utilize three sets of siderophores, acinetobactins (Fig. 1A, **1** and **2**) (14–17), fimsbactins (**3**) (18, 19), and baumannoferrins (**4**) (20) (Fig. 1A), although the presence of the genes responsible for their production and utilization differs across the strains (21, 22). Analysis of the genomes of various clinical isolates showed the prevalence of genes associated with acinetobactin biosynthesis and utilization, suggesting that this process would be the major Fe(III) assimilation mechanism and that acinetobactin likely serves as an important virulence factor for this pathogen (23). As detailed in our recent highlight article, the very significant progress made in the last decade has greatly enhanced our understanding of the physicochemical properties, iron-delivery function, and structure-function relationships of acinetobactin (24).

The structure of acinetobactin was originally assigned to be Oxa (Fig. 1A, **1**, often called “preacinetobactin”), featuring the oxazoline moiety at its core (14), but later biosynthetic studies by Walsh and coworkers revealed that although Oxa is the end product of the corresponding biosynthetic pathway, it can undergo spontaneous isomerization to yield thermodynamically stable Isox (**2**) (16, 17). However, no functional study to obtain information about their respective physiological functions was conducted at that time.

The use of multiple siderophores by a single bacterium is very common (Fig. 1B). These involve either multiple siderophore biosynthesis/utilization machineries or the exploitation of siderophores produced by other bacteria, often called xenosiderophores, through receptors with relaxed substrate specificity (25, 26). Unlike these two cases, the acinetobactin metabolism is unique, in that two different, yet structurally related, siderophore molecules are generated via isomerization; moreover, there is only limited information on the functional differences between two acinetobactins in regard to iron supply at infection sites. Unraveling the mode of acinetobactin utilization is anticipated to present a new model of multiple siderophore usage in bacterial iron assimilation.

Here, we present the results of biochemical investigation of the distinctive roles of the two acinetobactin isomers in terms of iron supply and virulence. First, Oxa was confirmed to be the preferred substrate for the outer membrane TonB-dependent transporter in the acinetobactin gene cluster. However, the machineries responsible for the cytoplasmic delivery from the periplasm appeared to recognize both isomers of acinetobactin. In addition, the two isomers were shown to be versatile metal chelators capable of binding with a variety of metal ions, not only iron. Importantly, Oxa was found to form a stable iron complex from which the iron cannot be displaced by Isox. This observation suggests that when two acinetobactin isomers compete for iron, the iron pool is likely to be channeled to Oxa. Finally, the observed capability of each acinetobactin to respond to the challenges imposed by host metal sequestering proteins well demonstrated the competency of both acinetobactin isomers to neutralize nutritional immune challenges. These new findings have led us to outline a new model that portrays distinctive roles for the two acinetobactin isomers in the context of the core Fe(III) supply chain of A. baumannii.

## RESULTS

### Disruption of the genes for acinetobactin uptake significantly compromises the growth of A. baumannii under iron-deficient conditions.

The genes responsible for acinetobactin uptake and utilization are clustered in the *bauDCEBA* operon (Fig. S1A) (27, 28). Sequence analysis of the corresponding gene products indicates that acinetobactin delivers iron intracellularly via a pathway commonly found in Gram-negative bacteria (29). Briefly, BauA, a TonB-dependent outer membrane receptor, is likely to serve as the primary gateway for the transport of iron-bound acinetobactin into the periplasm from the extracellular medium. A periplasmic siderophore binding protein, BauB, then captures the iron-acinetobactin complex, and subsequent interaction with an inner membrane permease complex composed of BauC, BauD, and BauE triggers the cytoplasmic transportation. During this process, BauE, a cytoplasmic ATPase component, provides the necessary energy. The mobilization of iron from acinetobactin was proposed to involve the action of BauF, a homolog of a flavin-dependent oxidoreductase, suggesting that iron release would involve reduction of Fe(III) to Fe(II), in conjunction with reduced affinity of acinetobactin (24, 30). In fact, during preparation of the manuscript, Valentino et al. published an article describing the structural and biochemical characterization of BauF, confirming this proposition (31).

10.1128/mBio.02248-21.3 FIG S1 Disruption of *bauB*, *bauD*, and *bauF*. (A) The structure of the acinetobactin gene cluster. The *bauDCEBA* operon responsible for acinetobactin uptake is colored in red, and the *bauF* gene involved in the iron mobilization inside the cytoplasm is colored in blue. (B) Schematic diagram of overlap extension PCR-mediated *bauB* disruption. The second single-crossover homologous recombination between the *bauE* genes on the chromosome (by sucrose counterselection) led to *bauB* gene deletion. The *bauD* and *bauF* mutants were generated using similar procedures. *sacB*, levansucrase-encoding gene; *cat*, chloramphenicol resistance gene. (C) PCR analysis of each wild-type and mutant strain generated by allelic exchange. Molecular size markers (1-kb DNA ladder) are indicated. WT, wild-type; Δ*bauB*, *bauB* mutant; Δ*bauD*, *bauD* mutant; Δ*bauF*, *bauF* mutant. Download FIG S1, PDF file, 0.2 MB. Copyright © 2021 Kim et al. 2021 Kim et al. https://creativecommons.org/licenses/by/4.0/ This content is distributed under the terms of the Creative Commons Attribution 4.0 International license.

To investigate the functional roles and substrate specificity of some of these cellular components, the *bauB*, *bauD*, and *bauF* genes of A. baumannii ATCC 19606 were individually disrupted to prepare the corresponding mutant strains (Fig. S1 and S2) (32). Previously, disruption of *bauA* and *basD*, a gene involved in the biosynthesis of acinetobactin, was found to cause significant growth suppression under iron-deficient conditions (32–34). Likewise, the effect of the newly mutated *bauB*, *bauD*, and *bauF* genes on bacterial growth in Luria-Bertani (LB) medium under iron-deficient conditions created by supplementation of 200 μM 2,2′-bipyridyl (DP) was examined (Fig. 2). In comparison with that of the wild type, the growth of the Δ*bauB* and Δ*bauD* mutant strains was significantly compromised in the iron-deficient medium, as in the case of the Δ*bauA* and Δ*basD* mutants, presumably because of the defects in the acinetobactin uptake (Fig. 2B). Interestingly, the growth of the Δ*bauF* mutant strain was not attenuated under the same conditions and was comparable to that of the wild type. This result suggests that, as opposed to the hypothesized role as a virulence factor (31), the function of BauF is not essential for mobilizing Fe(III) from the corresponding acinetobactin complex and is likely to be complemented by other oxidoreductases or biological reductants such as glutathione (30).

10.1128/mBio.02248-21.4 FIG S2 Verification of the A. baumannii mutant strains. (A) Schematic diagram of overlap extension PCR for single-copy complementation of the Δ*bauD*, Δ*bauB*, Δ*bauA*, and Δ*basD* mutant strains. P*_bauDCEBA_*, promoter of *bauD*, *bauC*, *bauE*, *bauB*, and *bauA*; P*_basD_*, promoter of *basD*; Int I and Int II, intergenic regions located between DJ41_RS05115 and DJ41_RS05120. (B) PCR analysis of the genomic DNA of the wild type (WT), the mutants (Δ*bauD*, Δ*bauB*, Δ*bauA*, and Δ*basD*), and the corresponding complemented strains (C-*bauD*, C-*bauB*, C-*bauA*, and C-*basD*) using Int01F and Int02R primers. Each of the genomic DNAs of the wild type and mutants produced a 2.2-kb amplicon, whereas the PCR using genomic DNA from the Δ*bauD*, Δ*bauB*, Δ*bauA*, and Δ*basD* complemented strains resulted in 4.4-kb, 4.5-kb, 5.8-kb, and 6.0-kb amplicons, respectively. (C and D) Growth of the A. baumannii ATCC 19606 WT and complemented mutant strains in the LB medium (C) without or (D) with 200 μM DP, confirming no occurrence of the polar effect during each gene disruption. The growth of each bacterial strain was measured using a 1-cm cuvette after 24 h of incubation at 37°C. Error bars represent the standard deviations of three independent experiments. Statistical significance was assessed by one-way ANOVA tests (ns, not significant). Download FIG S2, PDF file, 0.5 MB. Copyright © 2021 Kim et al. 2021 Kim et al. https://creativecommons.org/licenses/by/4.0/ This content is distributed under the terms of the Creative Commons Attribution 4.0 International license.

### BauA is the key gatekeeper to discriminate between Oxa and Isox during their cellular uptake.

With the prepared *bauA*, *bauB*, and *bauD* mutants, the roles and specificity of the corresponding gene products in the acinetobactin-mediated iron uptake were investigated based on the growth promotion assay. Previously, Wencewicz and coworkers examined the activity of the iron-preloaded, *holo*-acinetobactin isomers with several A. baumannii ATCC 19606 mutants lacking *bauA*, *bauB*, or *bauD* and observed that both acinetobactins could promote the growth of all mutants (33). In contrast, in our analogous study employing *apo*-acinetobactin isomers, we found that *apo*-Oxa was incapable of promoting the growth of the *bauA* mutant, whereas *apo*-Isox could (34, 35). Based on this observation, we proposed that the acinetobactin uptake machinery would be selective for Oxa over Isox. In fact, our finding appears to be consistent with a recent study by Naismith and coworkers characterizing the substrate specificity and the X-ray crystallographic structure of BauA (36). Nevertheless, the cause of seemingly different observations between us and the Wencewicz laboratory still calls for clarification to provide clues for distinguishing the functional difference between two acinetobactin isomers. For that matter, here, both *apo*- and *holo*-forms of each acinetobactin were subjected to a series of growth promotion assays under controlled identical conditions, as the main difference in the experimental setting between two laboratories appeared to be the iron-chelation state of acinetobactins.

**FIG 3:**
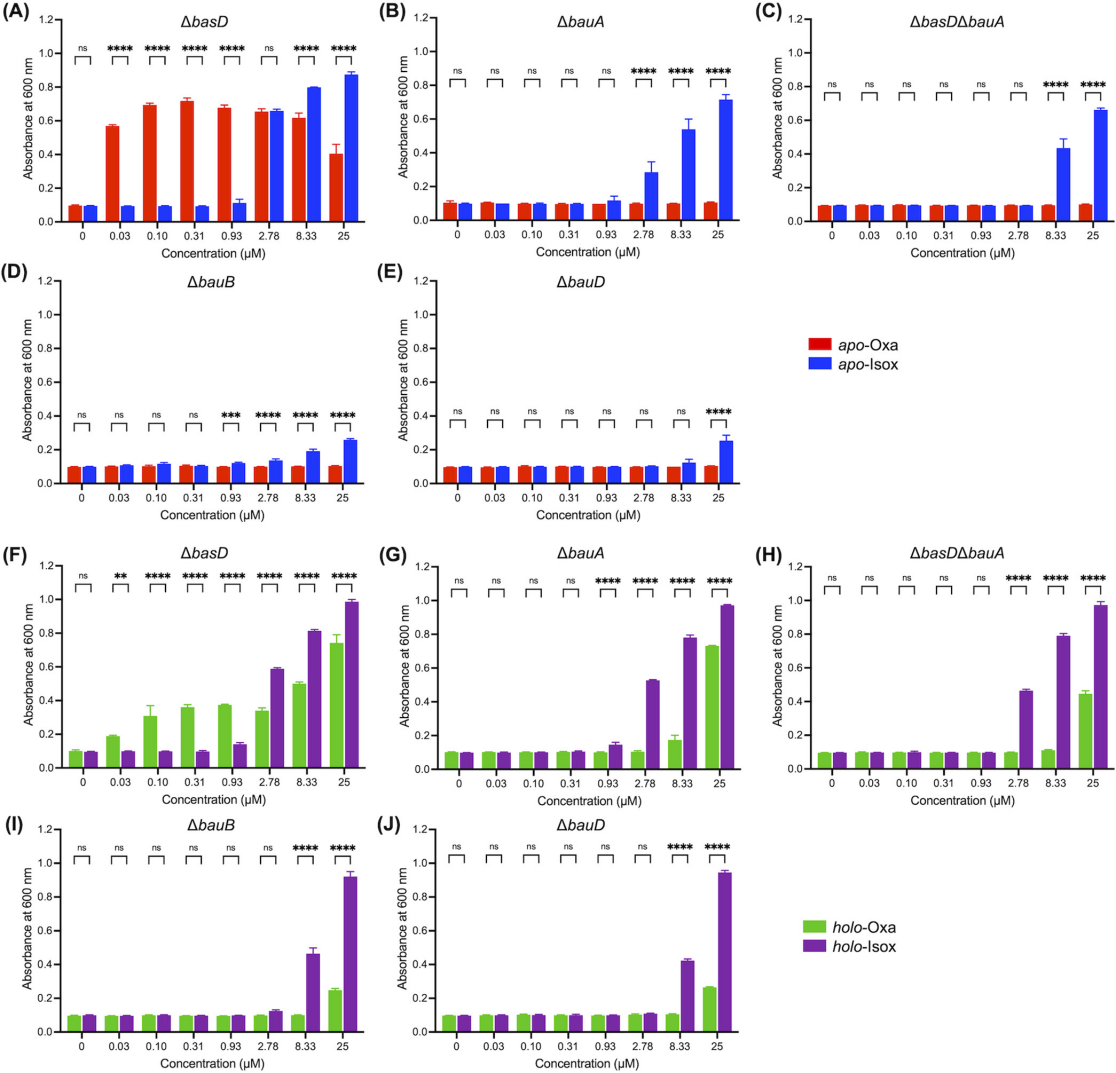
Growth-promoting activity assay results. (A to D) The *apo*-forms of acinetobactins were used to treat A. baumannii ATCC 19606 (A) Δ*basD*, (B) Δ*bauA*, (C) Δ*basD*Δ*bauA*, (D) Δ*bauB*, and (E) Δ*bauD* under iron-deficient conditions (LB medium with 200 μM DP). In addition, the respective mutants under iron-deficient conditions were treated with the *holo*-forms of acinetobactins (F to J). These assays were conducted on a 200-μl scale by using 96-well transparent microplates. After incubation of the strains at 37°C for 24 h (*apo*-forms) or 12 h (*holo*-forms), the absorbance at 600 nm was recorded by using a microplate reader. The time course growth curves over 24 h can be found in Fig. S3. Error bars represent the standard deviations of independent triplicate experiments. Statistical significance was assessed by two-way ANOVA tests (ns, not significant; **, *P < *0.01; ***, *P < *0.001; ****, *P < *0.0001).

First, the growth promotion assays were conducted with *apo*-acinetobactins. In this experimental setting, the amount of the iron-acinetobactin complex, formed *in situ*, is limited by the iron level in the medium. The results for A. baumannii Δ*bauA* and Δ*basD* were consistent with our previous observations (34, 35). Namely, *apo*-Oxa (Fig. 1A, **1**) could stimulate growth of A. baumannii Δ*basD* very effectively even at a concentration as low as 0.03 μM (Fig. 3A and Fig. S4A), while gradual attenuation of activity was observed in the high concentration range as reported in other studies probing the effects of *apo*-fimsbactin A and *apo*-anguibactin, a thermally stable surrogate of Oxa, on the A. baumannii growth (35, 37, 38). In contrast, the growth-promoting activity of *apo*-Isox (Fig. 1A, **2**) became noticeable only at ≥2.78 μM after 24 h of culturing (Fig. S4A). In the case of A. baumannii Δ*bauA* (Fig. 3B and Fig. S4B), no growth promotion was elicited by *apo*-Oxa within 24 h at any of the tested concentrations. However, the behavior with *apo*-Isox was similar to that observed for the Δ*basD* mutant strain as we previously reported (34, 35). As a control, a double knockout mutant, A. baumannii ATCC Δ*basD*Δ*bauA*, was generated. The growth promotion assay results on this mutant were similar to those of the *bauA* mutant (Fig. 3C and Fig. S4C). This confirms that the results for the single Δ*bauA* mutant are accurate and not distorted by endogenously biosynthesized acinetobactins. Then, the two other newly generated mutants, A. baumannii Δ*bauB* and Δ*bauD*, were subjected to the growth promotion assays (Fig. 3D and E and Fig. S4D and E). In general, the results were similar to those observed for the Δ*bauA* strain, but interestingly, the activity of *apo*-Isox was much lower in these mutants. This observation seems to suggest that the cytoplasmic transport of the iron-loaded Isox complex may somehow mediate the action of BauB and BauD. In fact, a previous report about the effective binding between BauB and Isox (apparent equilibrium dissociation constant [*K_D_*], 160 nM and 300 nM for *apo*- and *holo*-Isox, respectively) supports this possibility (39). Collectively, the growth promotion assay results using *apo*-acinetobactins demonstrated that the crossing of the iron-Oxa complex through the outer and inner membranes into the cytoplasm is principally dependent on the siderophore uptake channel composed by BauA, BauB, and a permease complex containing BauD. In the case of the iron-Isox complex, although its uptake through the outer membrane is less likely to involve the function of BauA, BauB and BauD appear to take some parts in its transport across the inner membrane.

10.1128/mBio.02248-21.6 FIG S4 Statistical analysis of the growth-promoting activity of acinetobactins under the iron-deficient conditions created by 200 μM DP. The bar graph results are essentially identical to those in Fig. 3. Statistical significance of the growth-promoting activity difference between each acinetobactin at the indicated concentration and the DMSO control was assessed by one-way ANOVA tests (ns, not significant; *, *P < *0.05; **, *P < *0.01; ***, *P < *0.001; ****, *P < *0.0001). Download FIG S4, PDF file, 0.5 MB. Copyright © 2021 Kim et al. 2021 Kim et al. https://creativecommons.org/licenses/by/4.0/ This content is distributed under the terms of the Creative Commons Attribution 4.0 International license.

10.1128/mBio.02248-21.5 FIG S3 Growth curves of the A. baumannii mutants treated with the indicated form of acinetobactin under iron-deficient conditions created by 200 μM DP. All experiments were conducted using the LB medium containing 200 μM DP. Error bars represent the standard deviations of independent triplicate experiments. Download FIG S3, PDF file, 0.6 MB. Copyright © 2021 Kim et al. 2021 Kim et al. https://creativecommons.org/licenses/by/4.0/ This content is distributed under the terms of the Creative Commons Attribution 4.0 International license.

The activity of each iron-preloaded *holo*-form was also investigated. In this case, the concentration of the iron-acinetobactin complex is not limited by the iron level of the medium, but it is, rather, directly proportional to the amount of the supplemented *holo*-acinetobactin. Treatment with *holo*-Isox resulted in overall similar trends to those with the corresponding *apo*-form but with a higher activity (Fig. 3F to J and Fig. S4F to J). Interestingly, however, *holo*-Oxa displayed growth-promoting activity for all *bau* mutants, unlike *apo*-Oxa, at high concentrations as reported by Wencewicz and coworkers (33). These observations seem to indicate that while the transporter machinery encoded in the *bauDCEBA* operon is designated to provide the major Oxa-mediated Fe(III) delivery pathway, the dependence on this system can be overridden by excess pool of *holo*-acinetobactin. Such cognate transporter-independent cellular uptake of *holo*-acinetobactins is likely to involve utilization of alternative TonB-dependent siderophore transporters encoded in the genome, such as PiuA and PirA related to the uptake of catechol-containing antibiotics, including BAL30072 (21, 40). Nevertheless, the physiological significance of the iron uptake via this alternative pathway is uncertain and likely to vary across the A. baumannii strains, considering the observed large strain-to-strain variation in the acinetobactin titer (0.5 to 48.8 μM) under laboratory culture conditions (41).

### Both acinetobactins are versatile metal chelators, not specific for iron.

Previous studies by us and the Wencewicz group have demonstrated that *apo*-Oxa undergoes isomerization readily to produce *apo*-Isox at a neutral pH (33, 34). Since the pH value of LB medium is approximately 7, it may be suspected that such an isomerization could have occurred under the growth promotion assay conditions. If this is the case, the observed growth-promoting activity difference between two acinetobactins would be hard to explain. However, it is worth noting previous reports that have demonstrated effective prevention of such isomerization by Oxa binding of Fe(III) (33, 36). The iron concentration of our typical LB preparation was 3.7 to 5.9 μM, and the 2:1 binding stoichiometry between Oxa and Fe(III) was well established (33, 34, 36). Thus, theoretically, *apo*-Oxa incubated in the LB medium could be resistant to isomerization up to 7.4 to 11.8 μM because of formation of *holo*-Oxa via Fe(III) complexation.

**FIG 4:**
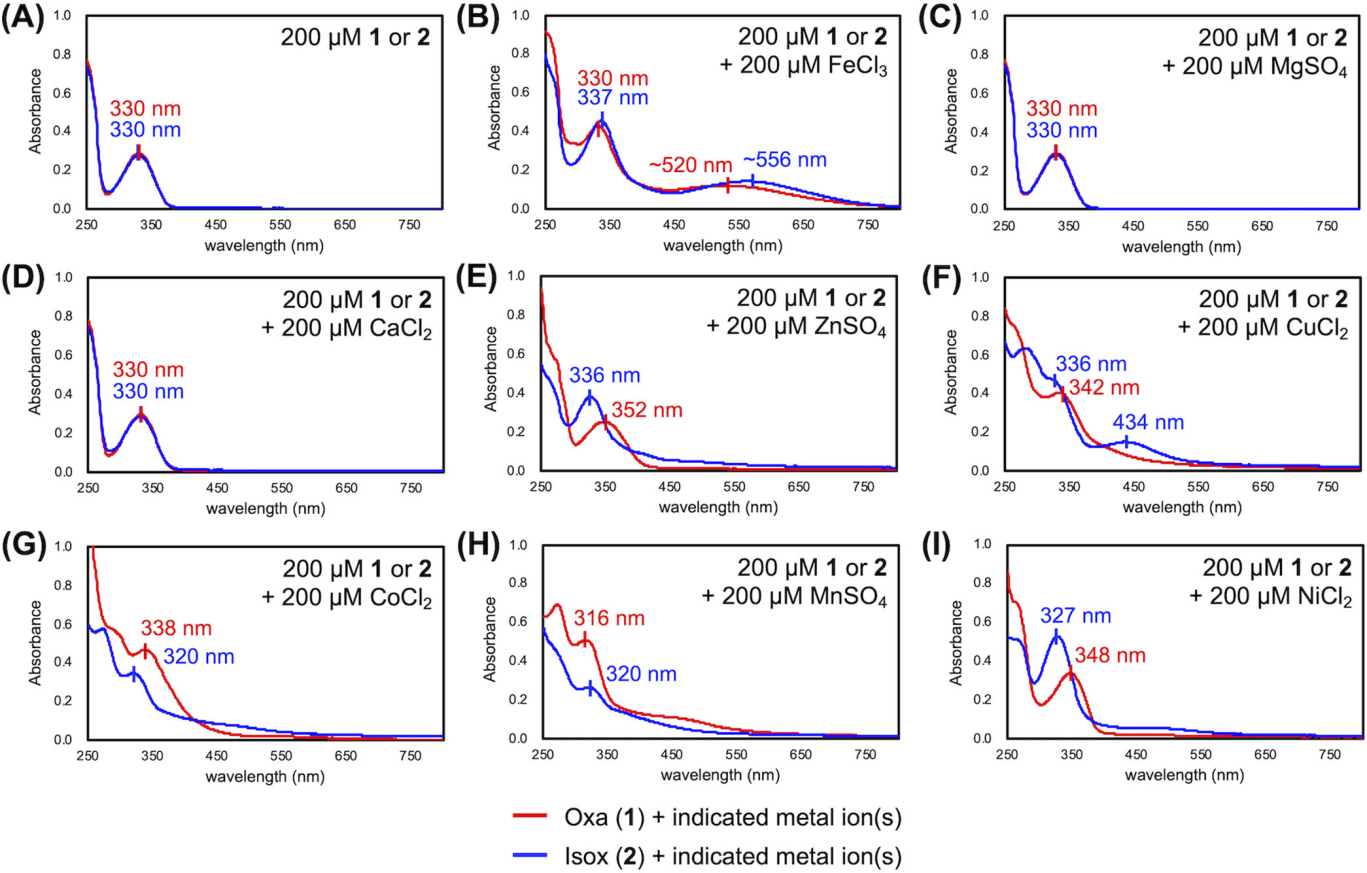
Analysis of the binding properties of acinetobactins for various metal ions. (A to I) The UV-visible absorption spectra of Oxa (1) and Isox (1) after 24 h of incubation in 100 mM Tris·HCl buffer (pH 7) at room temperature containing (A) no additive, (B) FeCl_3_, (C) MgSO_4_, (D) CaCl_2_, (E) ZnSO_4_, (F) CuCl_2_, (G) CoCl_2_, (H) MnSO_4_, and (I) NiCl_2_. The wavelengths of distinctive peaks are labeled.

To confirm whether Fe(III) chelation does indeed inhibit Oxa isomerization, UV-visible absorption spectroscopy experiments were conducted. Specifically, Oxa and Isox in pH 7 Tris buffer were treated with Fe(III), and the absorption spectra were compared after 24 h of incubation at 37°C. If Fe(III) chelation does inhibit the isomerization, the two acinetobactins are expected to display distinctive spectra. As shown in Fig. 4A, in the absence of any metal ion, *apo*-Oxa instantaneously isomerized, resulting in a spectrum identical to that of *apo*-Isox. In contrast, as shown in Fig. 4B, the supplementation of an equimolar amount of FeCl_3_ to *apo*-Oxa and *apo*-Isox afforded two spectroscopically different species. This observation clearly shows that the formation of *holo*-Oxa can effectively prevent its isomerization, even after prolonged incubation, confirming the inhibitory effect of Fe(III) chelation.

To extend this finding, the ability of each acinetobactin to interact with various noniron metal ions was investigated by application of the same protocol. The observation of indistinguishable spectra in the presence of Mg(II) or Ca(II) ions indicates that both acinetobactins are less likely to chelate these metal ions (Fig. 4C and D). In contrast, when *apo*-Oxa and *apo*-Isox were individually incubated with a number of biologically relevant transition metal ions, including Zn(II), Cu(II), Co(II), Mn(II), and Ni(II), discernible spectral profiles for the two acinetobactins emerged as a result of metal complexation in all cases (Fig. 4E to I). This metal complexation experiment not only shows that Oxa isomerization can be inhibited by various metal ions in addition to Fe(III) ions, but also reveals that both acinetobactins are indeed nonspecific, versatile metal chelators.

### Oxa forms a stable complex with Fe(III) whose iron is resistant to being displaced by Isox.

**FIG 5:**
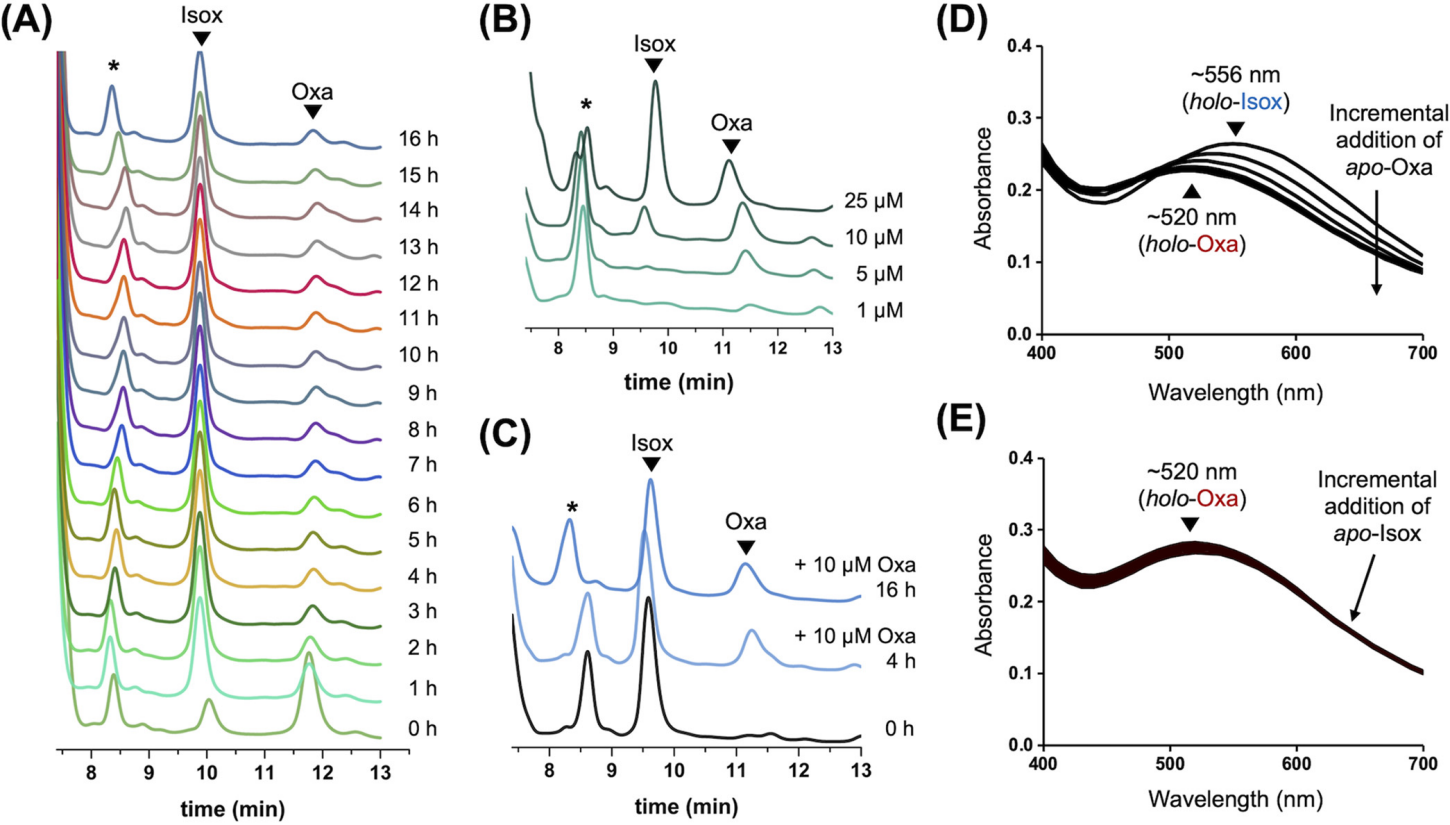
Analysis of the isomerization of Oxa to Isox in the presence of Fe(III) ions. (A) Time-course HPLC analysis tracing the isomerization of 25 μM Oxa in LB medium at 37°C. (B) HPLC chromatograms after 16 h of incubation for four different concentrations of *apo*-Oxa in LB medium at 37°C. (C) Time-course HPLC analysis of an LB medium solution preincubated with 25 μM *apo*-Isox for 1 h at 37°C and then treated with 10 μM *apo*-Oxa. (D and E) The absorption spectrum changes in the titration experiments in which (D) 0.1 to 1.0 equivalent of *apo*-Oxa was incrementally added to *holo*-Isox in 0.1 equivalent steps and (E) 0.1 to 1.0 equivalent of *apo*-Isox was incrementally added to *holo*-Oxa in 0.1 equivalent steps. All HPLC peaks were detected at 254 nm. It should be noted that our HPLC conditions with the eluents containing 0.1% trifluoroacetic acid (TFA) cannot distinguish *apo*-acinetobactin from the corresponding *holo*-form, likely as a result of Fe(III) dissociation by imidazole protonation under acidic pH conditions. The asterisk (*) indicates a peak originating from the LB medium.

After the suppression of Oxa isomerization by metal complexation was confirmed, a time course analysis based on high-performance liquid chromatography (HPLC) was conducted to identify the acinetobactin species present in the LB medium employed in the growth-promotion assay. As shown in Fig. 5A, when 25 μM Oxa (11.5 min) was added to LB medium at 37°C, isomerization to Isox (9.7 min) began occurring almost immediately (see the chromatogram marked “0 h”). Despite such rapid initial isomerization, a portion of Oxa was found to remain intact even after 16 h of incubation. Such stability of Oxa is likely to involve complexation of Fe(III) present in the LB medium.

To gather more information about the correlation between the presence of Fe(III) and Oxa stability, four different concentrations of *apo*-Oxa were mixed with the LB medium, and the mixtures were analyzed by HPLC after 16 h of incubation at 37°C. As shown in Fig. 5B, when 1 μM or 5 μM *apo*-Oxa was supplemented, no isomerization was observed, presumably due to complete formation of the iron complex. In contrast, upon excess use of *apo*-Oxa, isomerization was observed, while the degree of isomerization at 10 μM *apo*-Oxa treatment was much smaller than that with 25 μM *apo*-Oxa. Notably, the areas under the Oxa peak were nearly identical with 10 μM and 25 μM *apo*-Oxa. This result clearly shows that the amount of remaining Oxa is limited by the Fe(III) concentration in the medium, not by the concentration of supplemented *apo*-Oxa. In fact, the observed sustainability of Oxa is very intriguing, as it suggests that *apo*-Isox cannot displace Fe(III) from *holo*-Oxa. Otherwise, *apo*-Isox, generated by the addition of excess *apo*-Oxa, should have led to the release of *apo*-Oxa, with the accompanying rapid disappearance of the corresponding peak by isomerization.

To investigate Fe(III) displacement in the opposite direction, an additional experiment was conducted. A concentration of 25 μM *apo*-Isox was preincubated in LB medium for 1 h to allow effective Fe(III) complexation and complete depletion of mobile Fe(III) ions in the medium. The resulting solution was then exposed to 10 μM *apo*-Oxa to allow competition with *holo*-Isox for Fe(III). As shown in Fig. 5C, a substantial amount of Oxa persisted, regardless of the incubation time, which is consistent with efficient formation of *holo*-Oxa. Collectively, these results indicate that Fe(III) displacement under the current experimental conditions occurs only from Isox to Oxa, not the other way around. Indeed, direct evidence to support this unidirectional Fe(III) displacement was obtained by absorption spectroscopy experiments, in which the transformation of *apo*-Oxa to its *holo*-form via Fe(III) capture from *holo*-Isox was confirmed (Fig. 5D), whereas *holo*-Oxa was resistant to releasing Fe(III) ions in competition with *apo*-Isox (Fig. 5E). Such unidirectional Fe(III) displacement was unexpected because the apparent stability of both *holo*-acinetobactin complexes was reported to be comparable (apparent log *K*_Fe_, 27.4 ± 0.2 and 26.2 ± 0.1 for Oxa and Isox, respectively) (42). Although the resolution of these two seemingly contradictory observations is beyond the scope of this study, it is worth noting that a structural study by Naismith and coworkers has demonstrated that Oxa can chelate Fe(III) using its one hydroxyl group of the catechol, the hydroxy of the hydroxamate, and two nitrogen atoms of the oxazoline and imidazole rings (36). Such strong tetradentate iron coordination cannot be achieved by Isox because of the lack of the hydroxamate. Thus, the observed relative stability of the iron-Oxa complex over *holo*-Isox might have arisen from such a difference in the available mode of iron coordination between two acinetobactins.

### Both acinetobactins are competent metal scavengers capable of relieving nutritional challenge imposed by metal sequestering proteins.

The role of acinetobactin as a virulence factor is associated with its ability to compete against the host iron sequestering proteins transferrin (Tf) and lactoferrin (Lf). In fact, Ymamoto and coworkers have shown that acinetobactin can promote the growth of A. baumannii under iron-deficient conditions created by either Tf or Lf (41). However, their study was limited in that the two different acinetobactin forms had not been identified. In this study, to gain insights into the functional differences between the two acinetobactins in countering these host proteins, growth-promotion assays with individual acinetobactin forms were conducted. In addition, considering that both acinetobactins are also capable of chelating Zn(II), their interactions with a zinc sequestering protein, calprotectin (Cp), secreted by human immune cells as a defensive measure, were investigated (43).

**FIG 6:**
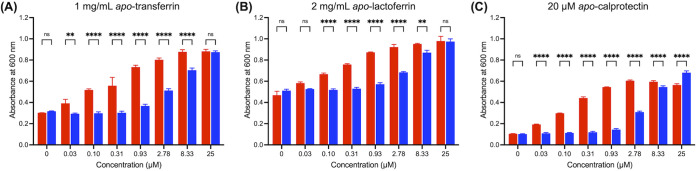
Growth-promoting activity assays in the presence of human metal sequestering proteins. Growth of A. baumannii Δ*basD* in the presence of different concentrations of *apo*-Oxa (**1**, red bar) or *apo*-Isox (**2**, blue bar) under iron-deficient conditions created by supplementation of *apo*-transferrin (1 mg/ml), *apo*-lactoferrin (2 mg/ml), or *apo*-calprotectin (20 μM) (Fig. 5). After incubation at 37°C for 12 h, the absorbance at 600 nm was recorded using a microplate reader. The time course growth curves over 24 h can be found in Fig. S6. The error bars represent the standard deviations of independent triplicate experiments. Statistical significance was assessed by two-way ANOVA tests (ns, not significant; **, *P < *0.01; ***, *P < *0.001; ****, *P < *0.0001).

Specifically, the A. baumannii Δ*basD* mutant growing in the presence of one of these proteins was exposed to each *apo*-acinetobactin to investigate the competition between them. As shown in Fig. 6 and Fig. S7, each *apo*-acinetobactin exhibited a similar growth-promoting activity to that observed when DP was used as a metal chelator (Fig. 3), no matter which metal sequestering protein was employed. This observation indicates that both acinetobactins are able to scavenge the metal ions withheld by Tf, Lf, and Cp and, thus, are capable of relieving the nutritional challenge imposed by these proteins; this result is consistent with the proposed roles of acinetobactins as virulence factors.

10.1128/mBio.02248-21.9 FIG S7 Statistical analysis on the growth-promoting activity of acinetobactins under the iron-deficient conditions created by metal sequestering proteins. The bar graph results are essentially identical to those in Fig. 6. Statistical significance of the growth-promoting activity difference between either *apo*-acinetobactin at the indicated concentrations and the DMSO control was assessed by one-way ANOVA tests (ns, not significant; *, *P < *0.05; **, *P < *0.01; ***, *P < *0.001; ****, *P < *0.0001). Download FIG S7, PDF file, 0.2 MB. Copyright © 2021 Kim et al. 2021 Kim et al. https://creativecommons.org/licenses/by/4.0/ This content is distributed under the terms of the Creative Commons Attribution 4.0 International license.

10.1128/mBio.02248-21.7 FIG S5 Growth of A. baumannii in the presence of *apo*-transferrin, *apo*-lactoferrin, or *apo*-calprotectin. (A and B) Growth curves of A. baumannii mutant Δ*basD* in the presence of (A) *apo*-transferrin and (B) *apo*-lactoferrin. (C to E) Growth curves of A. baumannii (C) WT, (D) Δ*basD*, and (E) Δ*bauA* in the presence of *apo*-calprotectin. Based on these results, the concentrations of *apo*-transferrin, *apo*-lactoferrin, and *apo*-calprotectin for growth promotion assays (Fig. 6) were selected to be 1 mg/ml, 2 mg/ml, and 20 μM, respectively. In addition, comparison of the growth curves in which the growth suppression effect of *apo*-calprotectin was more pronounced in the case of the *basD* and *bauA* mutants indicates that the acinetobactin mechanism may play some roles in ameliorating the metal deficiency created by calprotectin. The culture was conducted in LB medium at 37°C. Error bars represent the standard deviations of duplicate experiments. Download FIG S5, PDF file, 0.2 MB. Copyright © 2021 Kim et al. 2021 Kim et al. https://creativecommons.org/licenses/by/4.0/ This content is distributed under the terms of the Creative Commons Attribution 4.0 International license.

10.1128/mBio.02248-21.8 FIG S6 Growth curves of the A. baumannii Δ*basD* treated with either *apo*-Oxa or *apo*-Isox under iron-deficient conditions created by supplementation of the human metal sequestering proteins. All experiments were conducted using the LB medium containing 1 mg/ml *apo*-transferrin, 2 mg/ml *apo*-lactoferrin, or 20 μM *apo*-calprotectin. Error bars represent the standard deviations of independent triplicate experiments. Download FIG S6, PDF file, 0.3 MB. Copyright © 2021 Kim et al. 2021 Kim et al. https://creativecommons.org/licenses/by/4.0/ This content is distributed under the terms of the Creative Commons Attribution 4.0 International license.

### The growth promotion by each acinetobactin under challenge by Cp originates from successful intracellular delivery of iron, not zinc.

**FIG 7:**
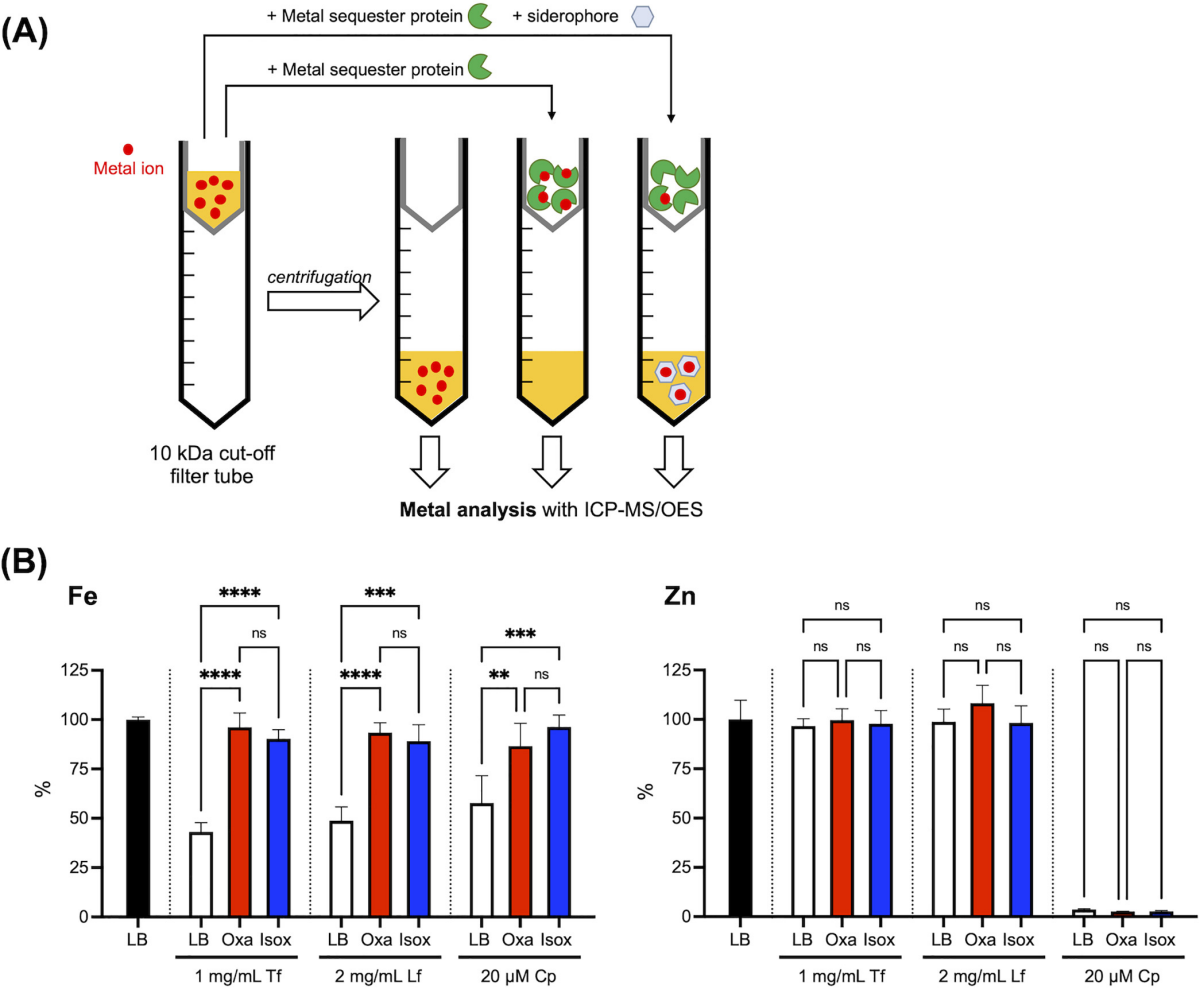
Metal sequestration assay. (A) Schematic of the metal sequestration assay. Briefly, in this assay, a metal sequestering protein and acinetobactin were added to the LB medium sequentially in that order with a 1-h interval for effective complexation of the former with metal ions in the medium before competition with the latter. After an additional 2 h of incubation at 37°C, each mixture was filtered through a membrane with 10-kDa cutoff, and the filtrate was then analyzed by ICP-OES/MS to quantify metals not withheld by the protein. The size of each acinetobactin is appreciably smaller than 10 kDa; therefore, any metal ion scavenged by this siderophore would be detected in the filtrate. (B) Metal sequestration assay results. All ICP-OES/MS signals were normalized with respect to the metal contents in the intact LB medium (100%). Error bars represent the standard deviations from independent triplicate experiments. Statistical significance was assessed by one-way ANOVA tests (ns, not significance; **, *P < *0.01; ***, *P < *0.001; ****, *P < *0.0001).

It has been well established that the primary functions of Tf and Lf are iron sequestration. In contrast, the physiological role of Cp was initially connected with its property of sequestering zinc and manganese from invading bacteria (44). However, recent reports have demonstrated that Cp is also capable of binding other metal ions, including iron (43, 45–48), indicating that its working mechanism in terms of the host defense may be more complex than originally conceived. In this regard, the observed growth-stimulating activity of acinetobactins under challenge by Cp is intriguing because it leads to questioning whether such activities originate from their ability to deliver iron or other metal ions, including zinc. To address this question, metal sequestration assays analyzing the metal content not withheld by Tf, Lf, or Cp based on inductively coupled plasma optical emission spectrometry or mass spectrometry (ICP-OES/MS) analysis were conducted as depicted in Fig. 7A (49).

A comparison of the ICP-OES/MS data for the intact LB medium and the Tf- or Lf-treated filtrates shows that both proteins are selective iron sequesters, as predicted, because iron is the only depleted metal species among the various transition metal ions, including zinc (Fig. 7B). When either 25 μM *apo*-Oxa or 25 μM *apo*-Isox was coincubated with each of these iron sequestering proteins, the iron levels in the filtrates noticeably increased, nearly reaching the level observed in intact LB, irrespective of the siderophore type. This result clearly shows that the iron-binding affinities of both acinetobactins are strong enough to provide A. baumannii with a powerful means of scavenging iron from Tf and Lf during infection. Analysis of the Cp-treated filtrate by ICP-OES/MS showed nearly complete depletion of zinc, as well as a significant reduction in the iron level (Fig. 7B), whereas the changes for other metal ions were negligible. Cp-treated LB was then challenged with the *apo*-form of each acinetobactin, and the metal contents in the resulting filtrates were analyzed. Interestingly, even though both acinetobactins are able to chelate Zn(II) in addition to Fe(III) (see Fig. 4), they appear to be able to scavenge only iron sequestered by Cp, not zinc. This result clearly shows that the observed growth promotion by acinetobactins in the presence of Cp originates from their siderophore activity and that neither of them functions as a zinc-delivery vehicle, often called a zincophore (25, 50).

## DISCUSSION

Regarding the differential roles of the two acinetobactin isomers in A. baumannii physiology, Shapiro and Wencewicz have proposed an intriguing model based on the pH-dependent chemical stability of Oxa involving its isomerization (33). This model suggests that Oxa would be the dominant isomer under acidic conditions, such as at infection sites, whereas Isox would be prevalent in neutral or basic environments. Here, we have disclosed several important new characteristics of acinetobactin utilization that complement the Wencewicz model. First, our growth promotion assays using *apo*-acinetobactins, in which the iron level is limited as at infection sites, have clearly demonstrated that Oxa is a much more efficient iron carrier than Isox. In addition, the ability of Oxa to deliver iron is likely to be functional even at pH 7 based on the observed effective protection of the Oxa isomerization upon complexation with Fe(III), suggesting that Oxa would be the primary siderophore for A. baumannii regardless of the pH of the medium.

In terms of the acinetobactin uptake mechanism, the actions of BauB and BauD-associated permease complex in addition to BauA were confirmed to be crucial for the efficient cellular uptake of the iron-Oxa complex. In contrast, while the translocation of the iron-Isox complex across the outer membrane appears to be independent of BauA, its cytoplasmic delivery, at least in part, seems to mediate BauB and the inner membrane permease complex. Indeed, it is consistent with a report by Naismith and coworkers that has shown an exclusive interaction of BauA with *holo*-Oxa, not *holo*-Isox, in the isothermal titration calorimetry experiments (36). These observations indicate that BauA plays a gatekeeping role in discriminating between Oxa and Isox, and it accounts for the higher iron delivery efficiency of the former. One caveat to this conclusion is that at least three *bauA* alleles exist across various A. baumannii isolates. Since the sequence homology among these gene products is not very high (ca. 60%, Fig. S8), it is possible that some BauA variants other than that of A. baumannii ATCC 19606 may have different substrate preferences. Additionally, the observed *bauCDEBA* operon-independent growth promotion by *apo*-Isox, *holo*-Isox, and *holo*-Oxa at their high concentrations is intriguing, while its physiological relevance is still in question. To explain this unexpected observation, we proposed the involvement of other transporters in the A. baumannii genome, such as PiuA and PirA (40), confirmation of which still awaits experimental validation.

10.1128/mBio.02248-21.10 FIG S8 Sequence comparison among three A. baumannii BauA variants. The sequences of three BauA variants from A. baumannii ATCC 17978, ATCC 19606, and AC12 were aligned using Clustal Omega, and the calculated identity matrix is shown as a table. Download FIG S8, PDF file, 0.5 MB. Copyright © 2021 Kim et al. 2021 Kim et al. https://creativecommons.org/licenses/by/4.0/ This content is distributed under the terms of the Creative Commons Attribution 4.0 International license.

Another new finding that distinguishes the two acinetobactin isomers was the stability of the *holo*-Oxa complex, from which iron cannot be displaced by *apo*-Isox. In stark contrast, iron bound by Isox could be readily captured by *apo*-Oxa. This feature, initially observed by HPLC analysis of the chemical identity of acinetobactin species in LB medium (Fig. 5A to C), seemed contradictory to the reportedly comparable apparent affinity of each acinetobactin for Fe(III) ions (42). However, a titration experiment in which each *holo*-acinetobactin complex was challenged with the other *apo*-isomer (Fig. 5D and E) provided unequivocal evidence to support unidirectional Fe(III) displacement solely from Isox to Oxa.

**FIG 8:**
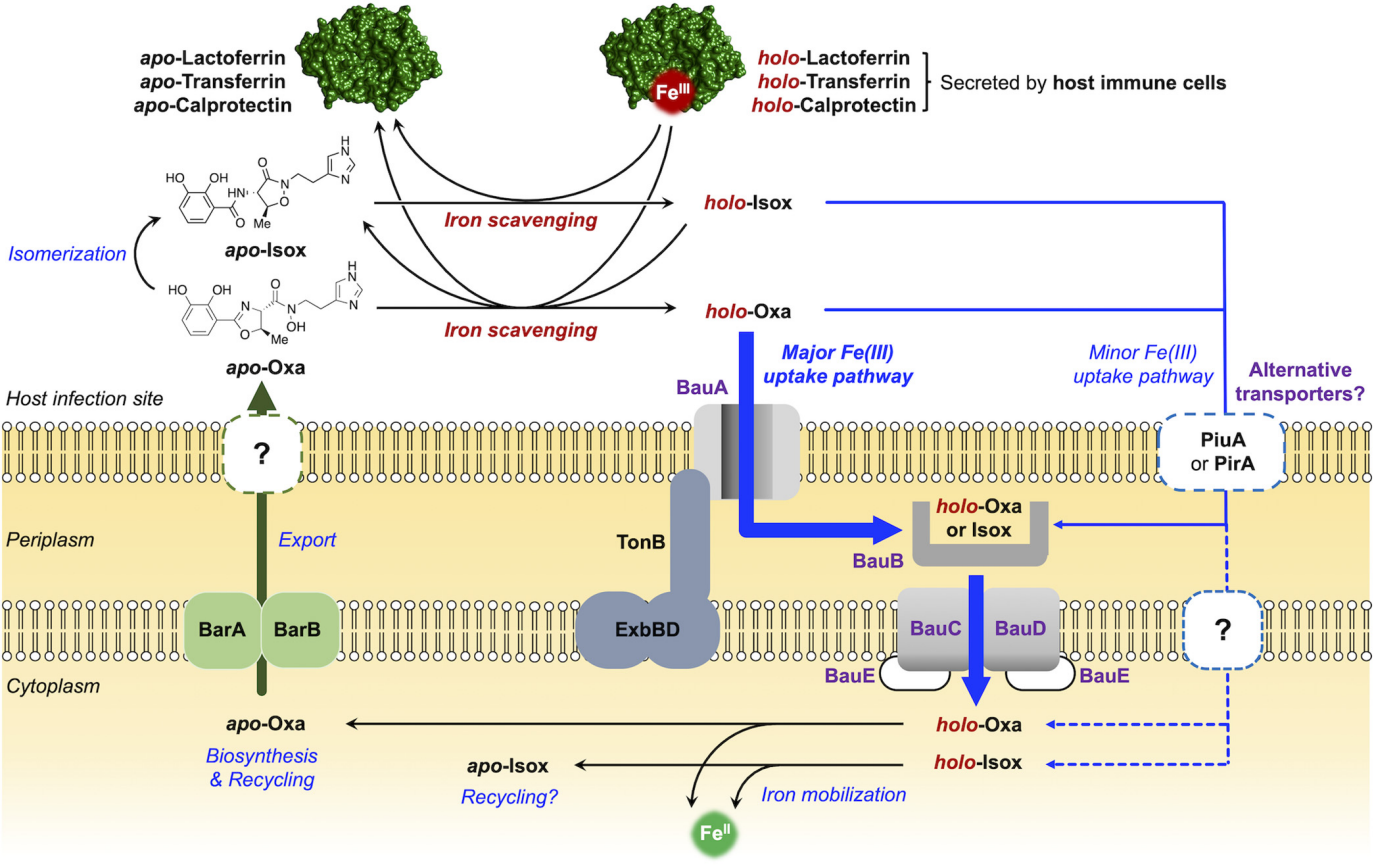
New proposed model for the acinetobactin-mediated iron acquisition and uptake mechanism of A. baumannii at infection sites. Three colors are used in arrows to indicate functional differences among individual processes; chemical reactions are in black, the export process is in green, and cellular uptake processes are in blue. Dashed arrows represent unconfirmed hypothetical processes.

Based on these distinguishing features of the two acinetobactin isomers, we propose a new model regarding their distinctive roles in sustaining the viability of A. baumannii during infection (Fig. 8). Upon invasion of the host, A. baumannii is immediately confronted with the limited supply of essential nutrients, including iron. The sequestration of iron by the host involves a number of different mechanisms, among which the metal sequestering proteins—Tf, Lf, and Cp—are responsible for restricting access to Fe(III) ions by A. baumannii. In response to such iron starvation challenges, A. baumannii begins biosynthesizing *apo*-Oxa via upregulation of the corresponding genes. Once exported out of the cell, *apo*-Oxa is faced with several possible paths, becoming a *holo-*form by pirating Fe(III) ions from iron sequestering proteins, being isomerized to *apo*-Isox, or staying in an intact *apo*-form, particularly in the acidic environments. At this stage, *holo*-Oxa is expected to effectively conduct the siderophore function of supplying A. baumannii with iron by utilizing the cognate transporter machinery initiated with the action of BauA. Recent structural analysis of BauA by Naismith and coworkers has shown the presence of a ternary heterocomplex composed of Fe(III), Oxa, and Isox in its substrate binding pocket upon soaking of *apo*-BauA with a 1:1 mixture of Fe(III) and Oxa (36), in which the residues therein have extensive interactions with Oxa chelating Fe(III) in a tetradentate fashion (see above). In this structure, the bidentate catechol moiety of Isox was found to occupy the remaining coordination sites of Fe(III). In contrast to Oxa, the majority of the Isox framework was solvent-exposed, and thus the interaction between Isox and the BauA residues was marginal. This pendant Isox ligand is likely to arise from the BauA-bound Fe(III)-Oxa_2_ complex via either on-site isomerization of the bidentate chelating Oxa or its replacement by *apo*-Isox present in the medium. Despite the observation of such a heterocomplex, the authors suggested the possibility that the catechol of Oxa could also occupy the bidentate coordination site of Fe(III) within the BauA substrate binding site. These results indicate that the actual formulation of *holo*-Oxa under physiological conditions could be either Fe(III)-Oxa-Isox or Fe(III)-Oxa_2_, whereas in either case, the tetradentate chelating Oxa would play the key motif to trigger the cellular uptake by BauA.

On the other hand, *apo*-Isox will also scavenge iron from the host, but the iron supply by the resulting *holo*-form is likely to be minor based on our growth promotion assay results, in which the activity of *holo*-Isox was only noticeable at high concentrations (Fig. 3). Alternatively, considering the intriguing irreversible capture of Fe(III) within *holo*-Isox by *apo*-Oxa (Fig. 5), it is conceivable that the function of Isox is to harvest iron from the environment and channel the collected iron pool to the available *apo*-Oxa. This proposition is consistent with the thermodynamics of Fe(III) binding; our data in Fig. 5 and 7 clearly indicate that when a limited quantity of Fe(III) is presented to a mixture of Oxa, Isox, and metal sequestering proteins, the equilibrium will eventually be funneled toward formation of *holo*-Oxa. Accordingly, it would be adequate to call Oxa the “principal siderophore” for A. baumannii in charge of the core Fe(III) supply chain, whereas Isox would serve as a “minor siderophore” as well as an “auxiliary iron collector.”

To the best of our knowledge, this proposed model depicted in Fig. 8 is unprecedented with respect to multiple siderophore utilization (Fig. 1B). When this is the case, the benefit of having an auxiliary iron collector like Isox is not yet certain in terms of A. baumannii virulence. In fact, the proposed role of Isox may be a mere serendipitous consequence, owing to the isomerization-prone characteristic of Oxa. However, it is also possible that the accompanying decrease in the level of *holo*-metal sequestering proteins may have some impact on the viability of the host cells surrounding A. baumannii, for example, through reduction in iron supply via cognate receptors (51, 52).

This study also revealed that both acinetobactins are versatile metal chelators (Fig. 4), not specific to iron, and the physiological relevance of this property was also of interest (53). However, ICP-OES/MS-based metal sequestration assays with calprotectin (Fig. 7) strongly imply that they do not function as zincophores. The functional relevance of the binding by acinetobactins of transition metals other than iron merits further investigation.

Since the first identification of Isox by Walsh and coworkers (16, 17), Oxa has often been referred to as “preacinetobactin,” whereas “acinetobactin” was coined for Isox. However, based on the experimentally verified efficient siderophore activity of Oxa as well as the well-established specificity of BauA for Oxa (36), Oxa deserves to regain its original designation as “acinetobactin.” Similarly, it would be logical to call Isox “isoacinetobactin,” because this term suitably represents both the chemical identity of Isox as an acinetobactin isomer and its auxiliary role in the A. baumannii iron supply chain.

Recently, Sheldon and Skaar reported *in vitro* and *in vivo* evidence to support the fact that acinetobactin, as a collective term, is the only siderophore among the other native siderophores, fimsbactins and baumannoferrins, required for virulence of A. baumannii (23). In conjunction with their conclusion, our results specifically underscore the significance of Oxa for A. baumannii infection. In this regard, we propose that future efforts to discover effective therapeutic means against A. baumannii focusing on the acinetobactin-based iron assimilation mechanism, for example, the development of an antibiotic delivery system, should be based on Oxa; the information presented in this work is anticipated to serve as an asset for such endeavors.

## MATERIALS AND METHODS

### Materials and apparatus.

All chemicals were purchased from Sigma-Aldrich (Missouri, USA), Acros Organics (New Jersey, USA), TCI Chemicals (Japan), Alfa-Aesar (Massachusetts, USA), AK Scientific (California, USA), or Daejung Chemicals & Metals (Republic of Korea), and they were used as received unless noted otherwise. The quality of the reagents was all BioReagent or at least ACS grade. Acinetobactin compounds (Oxa and Isox) were prepared as describe in our previous report (34). The oligonucleotides were purchased from Integrated DNA Technologies (IDT; Iowa, USA) or Macrogen (Republic of Korea), unless specified otherwise. Bacterial strains were obtained from the American Type Culture Collection (ATCC; Virginia, USA), the Korean Collection for Type Cultures (KCTC; Republic of Korea), or Addgene (Massachusetts, USA). Acinetobacter baumannii ATCC 19606, used as the primary test strain in this study, contains a gene cluster for biosynthesis and utilization of another siderophore called baumanoferrin (20), which may have some influences on the cellular growth under iron-deficient conditions. The Sanger sequencing was conducted via a service provided by Macrogen (Republic of Korea). The protein gel electrophoresis was conducted using a Mini-PROTEAN tetra vertical electrophoresis cell (Bio-Rad, California, USA). The DNA electrophoresis was conducted using a Mupid electrophoresis system (Nippon Genetics, Japan). The absorption spectroscopy experiment was conducted using an Epoch microplate spectrophotometer (BioTek, Vermont, USA) or Scinco S-3100 spectrophotometer (Republic of Korea) with a transparent 96-well or a quartz cuvette. The PCR were conducted using an AllInOneCycler (Bioneer, Republic of Korea). The high-performance liquid chromatography (HPLC) analysis was conducted using a Thermo Ultimate 3000 instrument (Thermo Fisher Scientific, Massachusetts, USA) equipped with a C_18_ reverse-phase column (Thermo Fisher Scientific; Acclaim 120 C_18_, 5 μm, 4.6 by 150 mm). All the HPLC solvents were filtered through a 0.45-μm membrane filter before use. The metal content analysis was conducted using either ICP-MS (NexION 300D; Perkin-Elmer, Massachusetts, USA) or ICP-OES (Agilent 730; California, USA).

### Overlap extension PCR-based gene disruption.

The strains and plasmids used in this study are shown in Table S1. Escherichia. coli and A. baumannii were cultured in LB broth or LB broth containing 1.5% (wt/vol) agar at 37°C. To maintain the plasmid in E. coli, the medium was supplemented with kanamycin (50 μg/ml) or chloramphenicol (20 μg/ml). The E. coli S17-1 λpir strains carrying each chimeric plasmid were employed as conjugal donors for A. baumannii strains. For conjugation, donor and recipient strains were cultured in LB broth until the late-log phase. Bacterial cells were then mixed at an equal ratio. Mixed bacterial cells were spotted onto an LB plate and then incubated for 12 h at 30°C. The medium was supplemented with kanamycin (30 μg/ml) and ampicillin (100 μg/ml) to select A. baumannii merodiploids. Plasmids with the antibiotic resistance cassette were eliminated from the bacterial chromosome on solid LB medium with 10% sucrose and without NaCl.

10.1128/mBio.02248-21.1 TABLE S1 Bacterial strains and plasmids used in this study. Download Table S1, PDF file, 0.2 MB. Copyright © 2021 Kim et al. 2021 Kim et al. https://creativecommons.org/licenses/by/4.0/ This content is distributed under the terms of the Creative Commons Attribution 4.0 International license.

### Complementation of the *bauD*, *bauB*, *bauA*, and *basD* mutants.

For single-copy complementation of the *bauD*, *bauB*, *bauA*, and *basD* mutants, DNA fragments, in which each of the targeted genes with its promoters was combined with *rrnB* terminator, were inserted *in vitro* into an intergenic region located in the downstream region of a gene (DJ41_RS05115) encoding hypothetical protein by overlap extension PCR (Fig. S2A) (54). For example, the *bauD* coding region with its native promoter and the intergenic regions I and II of the downstream were amplified from the genomic DNA of A. baumannii ATCC 19606 using the primer pairs C-BauDF/C-BauDR, Int01F/Int01R, and Int02F/Int02R, respectively (Table S2). To prevent effects on the expression of genes adjacent to the insertion loci, *rrnB* terminator was amplified using a primer pair R1/R2 (Table S2) and pSE380 as a template. *nptI* was amplified from the pUC4K. In particular, the primers C-BauDF/C-BauDR were designed to contain 25 additional nucleotides at their 5′ ends that are homologous to the downstream and upstream regions of the *rrnB* terminator, respectively. The primers R1 and R2 carry 25 additional nucleotides at their 5′ ends that are homologous to the intergenic regions I and II, respectively. The primer Int02R was also designed to contain an additional 20 nucleotides at its 5′ ends that are homologous to the kanamycin resistance cassette. The six PCR products obtained from the first PCR amplification were mixed at equimolar concentrations and subjected to overlap extension PCR using the primers Int01F/U2. The DNA fragments generated by overlap extension PCR were cloned into the FspI-digested pHKD01 to yield pOH1053 (Table S1). The conjugation and isolation of the transconjugants were accomplished as described in our previous report (32). Insertion of the targeted DNA fragment was confirmed by PCR analysis (Fig. S2B). In a similar way, each of the single-copy complemented strains (Table S1) was constructed and then confirmed by PCR analysis.

10.1128/mBio.02248-21.2 TABLE S2 Oligonucleotides used in this study. Download Table S2, PDF file, 0.2 MB. Copyright © 2021 Kim et al. 2021 Kim et al. https://creativecommons.org/licenses/by/4.0/ This content is distributed under the terms of the Creative Commons Attribution 4.0 International license.

### Growth assay under iron-deficient conditions.

A single colony was picked from a fresh Luria-Bertani (LB) agar plate overlaid with the A. baumannii strain of interest and was then used to inoculate 5 ml of LB medium. After incubation at 37°C with agitation at 200 rpm overnight in a shaking incubator, this solution was diluted with LB broth either with or without 200 μM 2,2′-bipyridyl (DP) in a 1:200 dilution. Each culture solution was then incubated at 37°C with agitation at 200 rpm for 24 h, followed by measurement of the absorption at 600 nm.

### Growth promotion assay using acinetobactin under iron-deficient conditions.

A single colony was picked from a fresh Luria-Bertani (LB) agar plate overlaid with the A. baumannii mutant strain of interest and was then used to inoculate 5 ml of LB medium. After incubation at 37°C with agitation at 200 rpm overnight in a shaking incubator, the medium of this solution was exchanged with 5 ml fresh LB medium, and then this suspension was diluted with LB broth containing 200 μM 2,2′-bipyridyl (DP), 1 mg/ml *apo*-transferrin (Sigma-Aldrich; catalog [cat.] no. T2252), 2 mg/ml *apo*-lactoferrin (Sigma-Aldrich; cat. no. L4040), or 20 μM *apo*-calprotectin (55) to reach an optical density at 600 nm (OD600) if ca. 0.05. A 196-μl aliquot of the diluted culture was mixed with a 4-μl aliquot of acinetobactin dimethyl sulfoxide (DMSO) stock solution (either *apo*- or *holo*-form) whose concentrations were adjusted to make the designated final concentrations indicated in Fig. 3 and 6 in a sterile Greiner Bio-One 96-well microplate (Austria). The microplate was incubated at 37°C with agitation, and the OD600 values were recorded every hour using a BioTek Epoch 2 microplate reader. All measurements were made using triplicate biological samples, and the mean values were used for plotting the results, in which the error bars indicate the standard deviation.

### Metal binding assay based on the absorption spectroscopy.

To a 100-mM Tris·HCl buffer at pH 7 was added each metal ion stock solution prepared by dissolving the salt (FeCl_3_, MgSO_4_, CaCl_2_, ZnSO_4_, CuCl_2_, CoCl_2_, and MnSO_4_) in deionized water to adjust the final concentration of the metal ion to be 200 μM. Then, either *apo*-Ab-Oxa or *apo*-Ab-Isox DMSO stock solution was added to this solution (1 ml) to the final concentration of 200 μM. After thorough vortexing, each mixture was incubated for 24 h at 37°C until the spectrum from 250 nm to 800 nm for each mixture was recorded using a BioTek Epoch 2 microplate reader.

### High-performance liquid chromatography (HPLC) analysis.

For HPLC analysis, a Thermo Ultimate 3000 device equipped with a C_18_ reverse-phase column (Thermo Fisher Scientific; Acclaim 120 C18, 5 μm, 4.6 by 150 mm) was employed. The eluent solvents were 0.1% trifluoroacetic acid (TFA)-containing deionized water (solvent A) and 0.1% TFA-containing acetonitrile (solvent B). Each solvent was filtered through a 0.45-μM membrane filter to remove any small particulate before use. Oxa and Isox were analyzed by isocratic elution of 13% solvent B for 15 min and absorption detection at 254 nm. The retention times of the peaks corresponding to these compounds were 11.5 min and 9.7 min, respectively.

### Competition between two acinetobactin isomers for iron binding.

For investigation of the capability of *apo*-acinetobactin to displace iron chelated by the other acinetobactin isomer, 200 μM *apo*-acinetobactin was mixed with a 100 mM Tris·HCl buffer (pH 7) containing 100 μM FeCl_3_ (ligand:iron = 2:1), and the resulting mixture was allowed to stand for 1 h at 37°C for complete formation of the corresponding *holo*-form. Then, the *apo*-form of the other acinetobactin isomer was incrementally added to this mixture and incubated for 30 min at 37°C to allow enough time for both acinetobactin isomers to compete with each other for iron binding. The absorption spectrum was recorded 30 min after every addition using a BioTek Epoch 2 spectrometer.

### Metal sequestration assay using ICP-MS and ICP-OES.

The schematic for this assay is depicted in Fig. 7A. First, the LB broth was incubated with one of the metal sequestering proteins for 1 h (1 mg/ml transferrin, 2 mg/ml lactoferrin, 20 μM calprotectin) at 37°C with gentle agitation to allow effective metal sequestration. Half of this solution was aliquoted to be mixed with 5 μM either *apo*-Oxa or *apo*-Isox, and the resulting mixture was incubated for 2 h at 37°C with gentle agitation to allow enough time for the acinetobactin molecule to scavenge metal ions withheld by a metal sequestering protein. Each sample, either supplemented with acinetobactin or not, was filtered through a 10-kDa cutoff membrane, and the filtrate was then analyzed using the inductively coupled plasma optical emission spectrophotometer (730 ICP-OES; Agilent, USA) or mass spectrometer (NexION 300D; Perkin-Elmer, USA) to measure the contents of the metal ions. As a control, a simple LB broth solution was analyzed in an analogous manner. The changes in the metal ion level relative to the LB broth control were only discernible for iron and zinc, and therefore only those data were included in Fig. 7B.

### Statistical analyses.

Statistical significance was assessed with either one-way or two-way analysis of variance (ANOVA) tests as indicated using GraphPad Prism V9.
