# Supplementary material for: Distinctive Roles of Two Acinetobactin Isomers in Challenging Host Nutritional Immunity
Source: mBio. 2021 Sep 14;12(5):e02248-21. doi: 10.1128/mBio.02248-21 (PMC8546848; doi:10.1128/mBio.02248-21)
Supplement: FIG S1 [file mbio.02248-21-sf001.pdf]

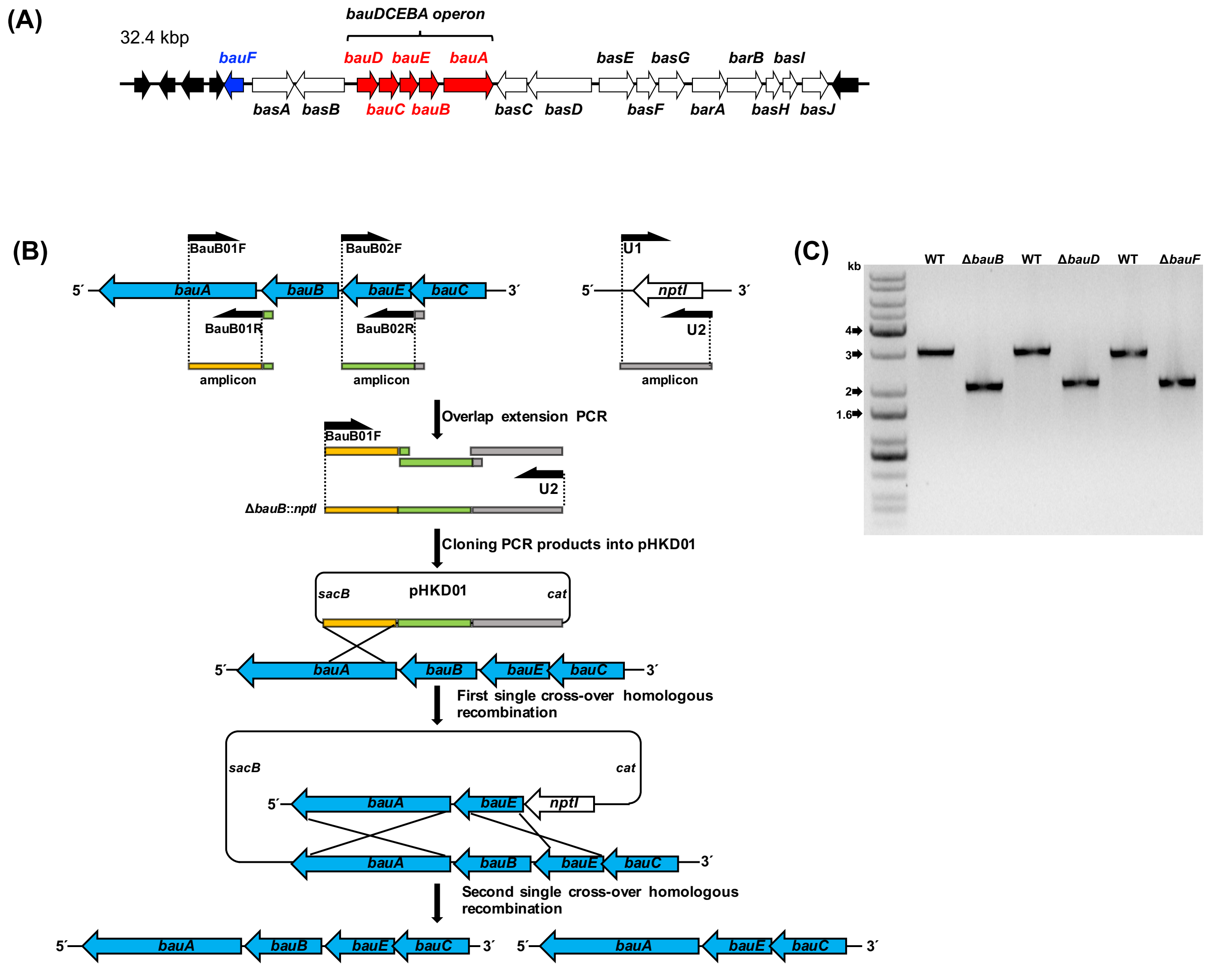

Fig. S1. Disruption of *bauB*, *bauD*, and *bauF*.

(A) The structure of the acinetobactin gene cluster. The *bauDCEBA* operon responsible for acinetobactin uptake is colored in red, and the *bauF* gene involved in the iron mobilization inside the cytoplasm is colored in blue. (B) Schematic diagram of overlap extension PCR-mediated *bauB* disruption. The second single-crossover homologous recombination between the *bauE* genes on the chromosome (by sucrose counter-selection) led to *bauB* gene deletion. The *bauD* and *bauF* mutants were generated using similar procedures. *sacB*, levansucrase-encoding gene; *cat*, chloramphenicol resistance gene. (C) PCR analysis of each wild-type and mutant strain generated by allelic exchange. Molecular size markers (1-kb DNA ladder) are indicated. WT, wild-type; Δ*bauB*, *bauB* mutant; Δ*bauD*, *bauD* mutant; Δ*bauF*, *bauF* mutant.
