# Supplementary material for: Distinctive Roles of Two Acinetobactin Isomers in Challenging Host Nutritional Immunity
Source: mBio. 2021 Sep 14;12(5):e02248-21. doi: 10.1128/mBio.02248-21 (PMC8546848; doi:10.1128/mBio.02248-21)
Supplement: FIG S2 [file mbio.02248-21-sf002.pdf]

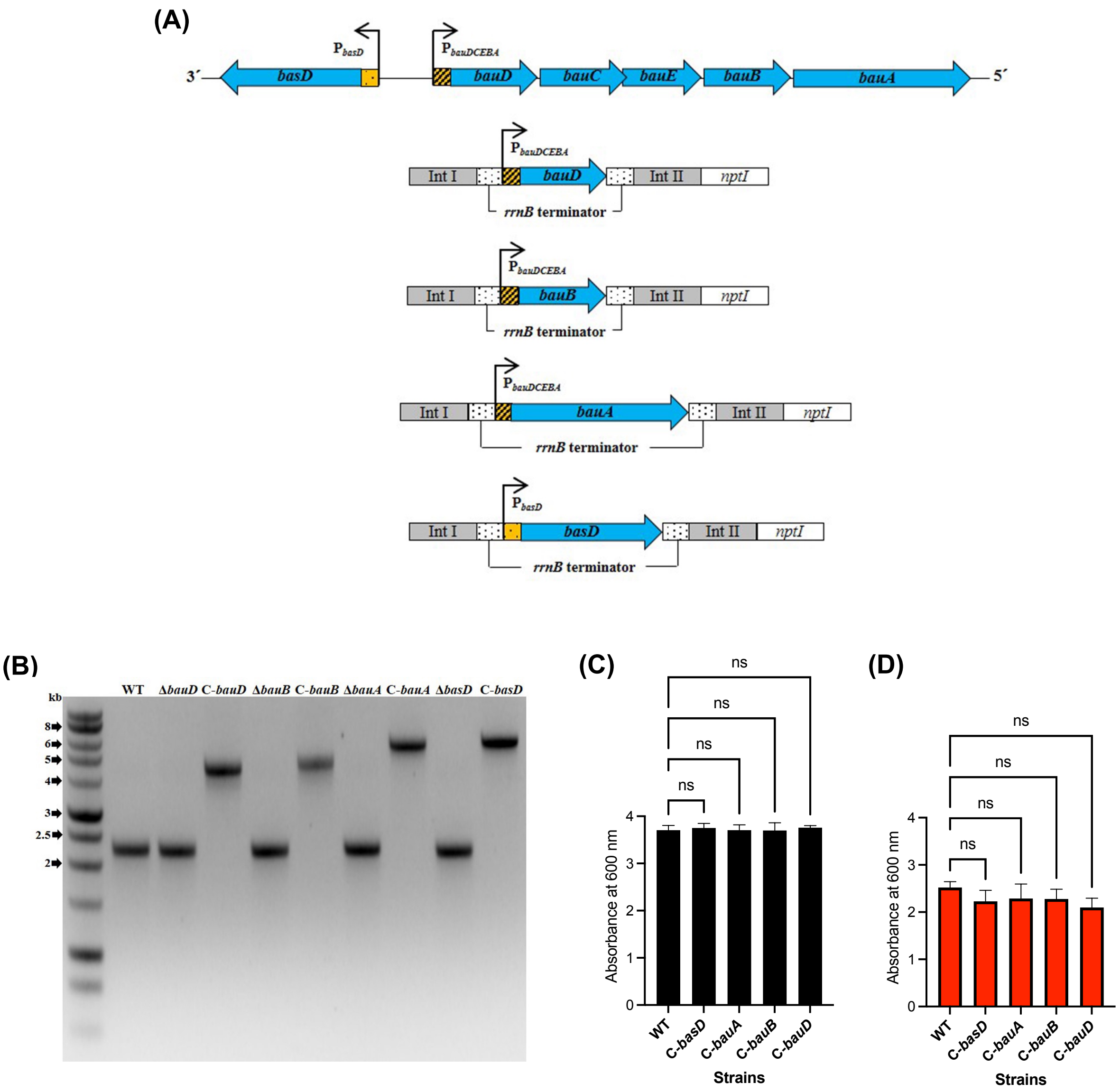


**Fig. S2. Verification of the *A. baumannii* mutant strains.**

(A) Schematic diagram of overlap extension PCR for single copy complementation of the Δ*bauD*, Δ*bauB*, Δ*bauA*, and Δ*basD* mutant strains. $P_{bauDCEBA}$, promoter of *bauD*, *bauC*, *bauE*, *bauB*, and *bauA*; $P_{basD}$, promoter of *basD*; Int I and Int II, intergenic regions located between DJ41_RS05115 and DJ41_RS05120. (B) PCR analysis of the genomic DNA of the wild-type (WT), mutants (Δ*bauD*, Δ*bauB*, Δ*bauA*, and Δ*basD*), and the corresponding complemented strains (C-*bauD*, C-*bauB*, C-*bauA*, C-*basD*) using Int01F and Int02R primers. Each of the genomic DNA of wild-type and mutants produced a 2.2-kb amplicon, whereas the PCR using genomic DNA from the Δ*bauD*, Δ*bauB*, Δ*bauA*, and Δ*basD* complemented strains resulted in 4.4-kb, 4.5-kb, 5.8-kb, and 6.0-kb amplicons, respectively. Growth of the *A. baumannii* ATCC 19606 WT and complemented mutant strains in the LB medium (C) without or (D) with 200 μM DP, confirming no occurrence of the polar effect during each gene disruption. The growth of each bacterial strain was measured using a 1 cm cuvette after 24 h incubation at 37 ºC. Error bars represent the standard deviations of three independent experiments. Statistical significance was assessed by one-way ANOVA tests (ns: not significant).
