# Supplementary material for: Distinctive Roles of Two Acinetobactin Isomers in Challenging Host Nutritional Immunity
Source: mBio. 2021 Sep 14;12(5):e02248-21. doi: 10.1128/mBio.02248-21 (PMC8546848; doi:10.1128/mBio.02248-21)
Supplement: FIG S4 [file mbio.02248-21-sf004.pdf]

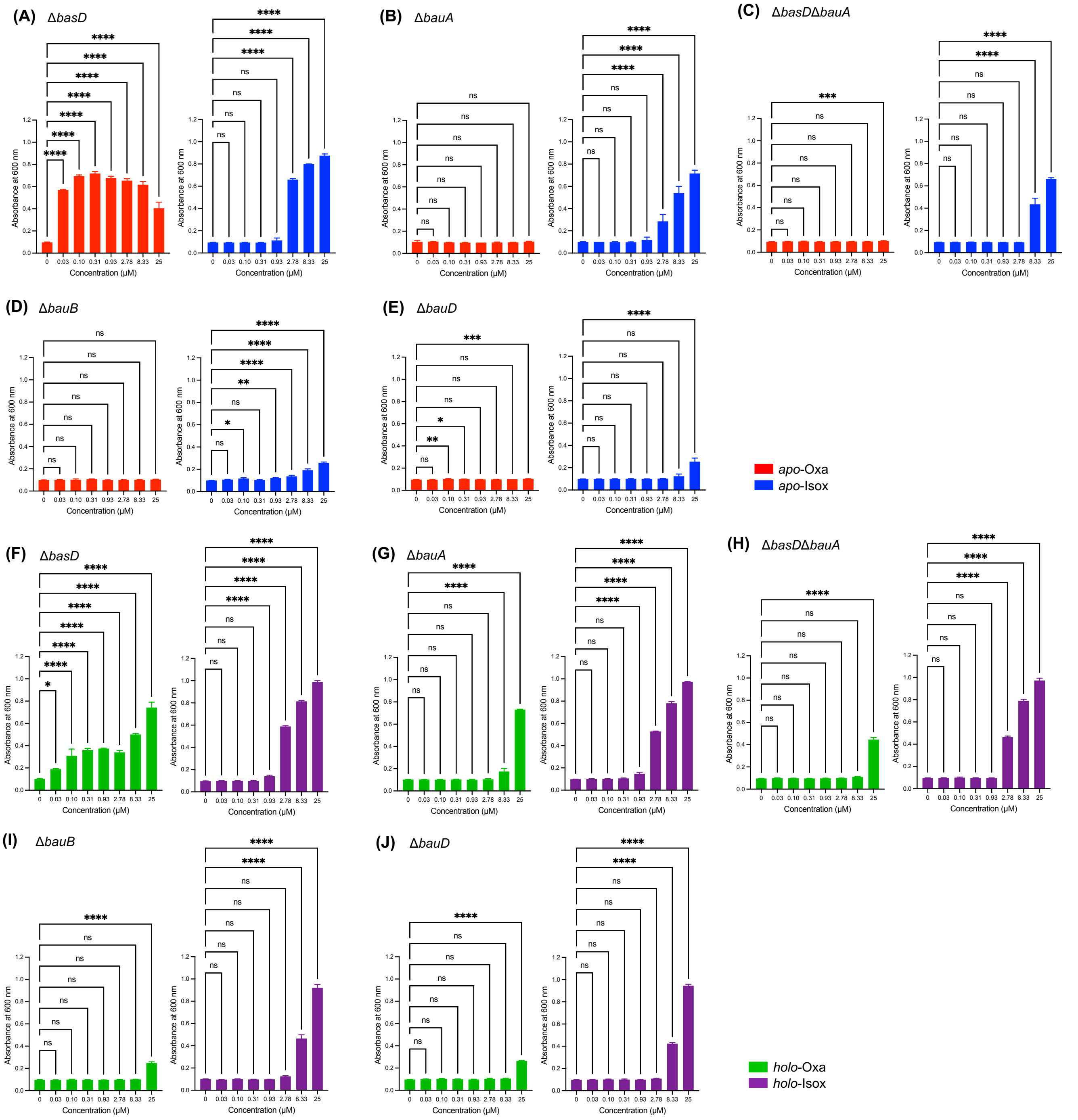

Fig S4. Statistical analysis on the growth promoting activity of acinetobactins under the iron-deficient conditions created by 200 μM DP.

The bar graph results are essentially identical to those in Fig. 3. Statistical significance of the growth promoting activity difference between each acinetobactin at the indicated concentration and the DMSO control was assessed by one-way ANOVA tests (ns: not significant, *: $p < 0.05$, **: $p < 0.01$, ***: $p < 0.001$, and ****: $p < 0.0001$).
