# Supplementary material for: Distinctive Roles of Two Acinetobactin Isomers in Challenging Host Nutritional Immunity
Source: mBio. 2021 Sep 14;12(5):e02248-21. doi: 10.1128/mBio.02248-21 (PMC8546848; doi:10.1128/mBio.02248-21)
Supplement: FIG S3 [file mbio.02248-21-sf003.pdf]

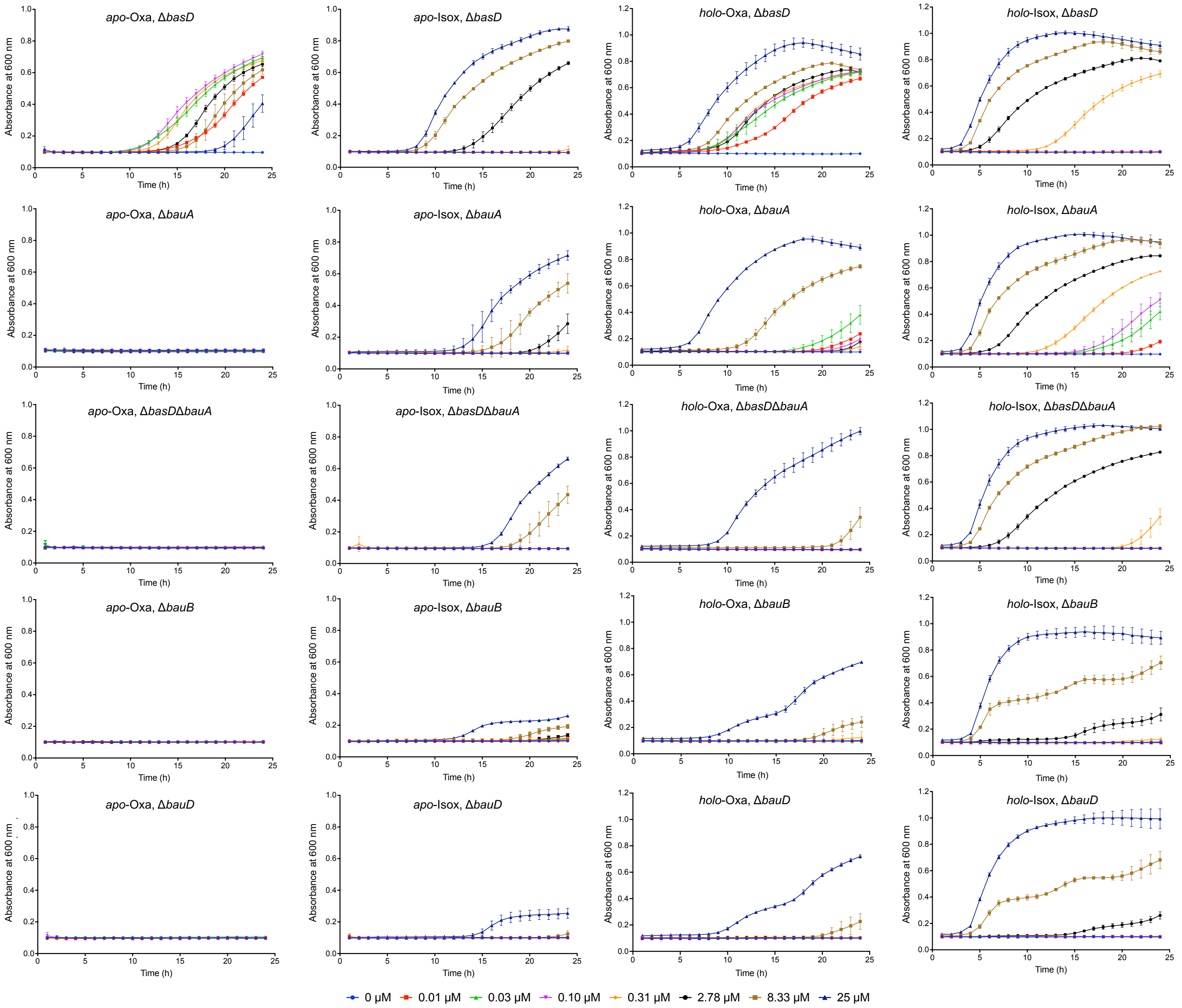

Fig S3. Growth curves of the *A. baumannii* mutants treated with the indicated form of acinetobactin under iron-deficient conditions (LB medium containing 200 μM DP).

All experiments were conducted using the LB media containing 200 μM DP. Error bars represent the standard deviations of independent triplicate experiments.
