# Supplementary material for: Distinctive Roles of Two Acinetobactin Isomers in Challenging Host Nutritional Immunity
Source: mBio. 2021 Sep 14;12(5):e02248-21. doi: 10.1128/mBio.02248-21 (PMC8546848; doi:10.1128/mBio.02248-21)
Supplement: FIG S7 [file mbio.02248-21-sf007.pdf]

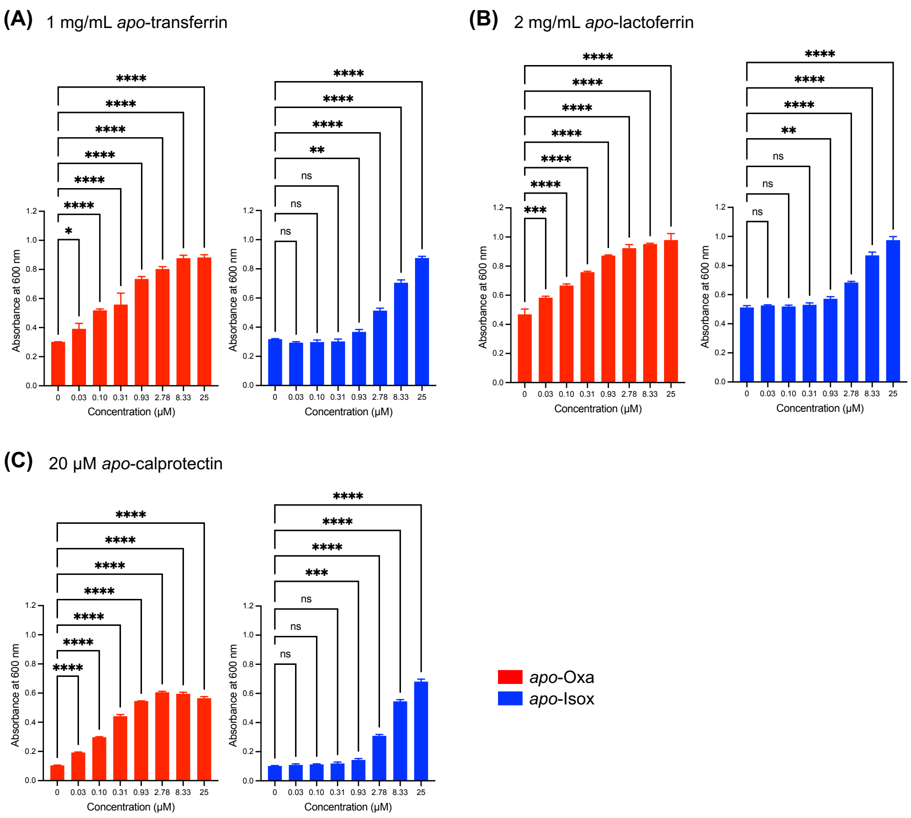

Fig S7. Statistical analysis on the growth promoting activity of acinetobactins under the iron-deficient conditions created by metal sequestering proteins.

The bar graph results are essentially identical to those in Fig. 6. Statistical significance of the growth promoting activity difference between either *apo*-acinetobactin at the indicated concentrations and the DMSO control was assessed by one-way ANOVA tests (ns: not significant, *: $p < 0.05$, **: $p < 0.01$, ***: $p < 0.001$, and ****: $p < 0.0001$).
