# Supplementary material for: Distinctive Roles of Two Acinetobactin Isomers in Challenging Host Nutritional Immunity
Source: mBio. 2021 Sep 14;12(5):e02248-21. doi: 10.1128/mBio.02248-21 (PMC8546848; doi:10.1128/mBio.02248-21)
Supplement: FIG S5 [file mbio.02248-21-sf005.pdf]

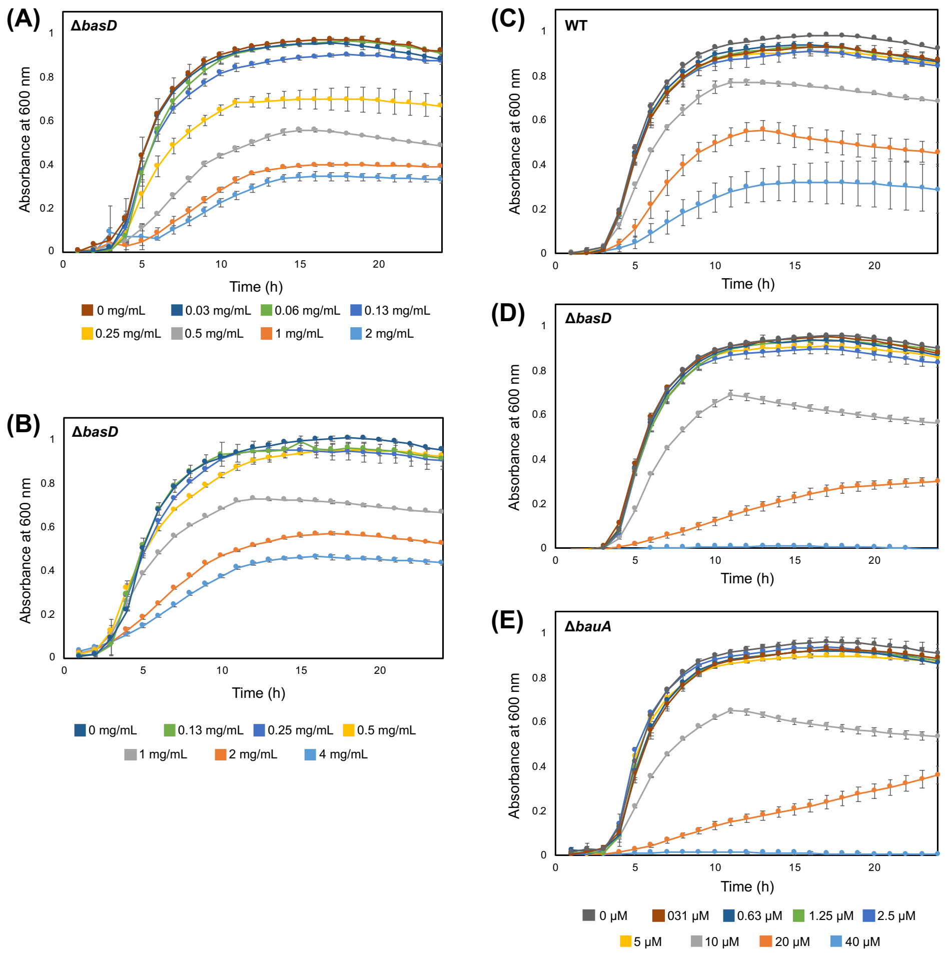


**Fig. S5. Growth of the *A. baumannii* in the presence of *apo*-transferrin, *apo*-lactoferrin, or *apo*-calprotectin**

Growth curves of *A. baumannii* mutant Δ*basD* in the presence of (A) *apo*-transferrin and (B) *apo*-lactoferrin. Growth curves of *A. baumannii* (C) WT, (D) Δ*basD*, and (E) Δ*bauA* in the presence of *apo*-calprotectin. Based on these results, the concentrations of *apo*-transferrin, *apo*-lactoferrin, and *apo*-calprotectin for growth promotion assays (Fig. 6) were selected to be 1 mg/mL, 2 mg/mL, and 20 µM, respectively. In addition, comparison of the growth curves in which the growth suppression effect of *apo*-calprotectin was more pronounced in the case of the *basD* and *bauA* mutants indicates that the acinetobactin mechanism may play some roles in ameliorating the metal deficiency created by calprotectin. The culture was conducted in LB media at 37 ºC. Error bars represent the standard deviations of duplicate experiments.
