# Supplementary material for: Distinctive Roles of Two Acinetobactin Isomers in Challenging Host Nutritional Immunity
Source: mBio. 2021 Sep 14;12(5):e02248-21. doi: 10.1128/mBio.02248-21 (PMC8546848; doi:10.1128/mBio.02248-21)
Supplement: FIG S6 [file mbio.02248-21-sf006.pdf]

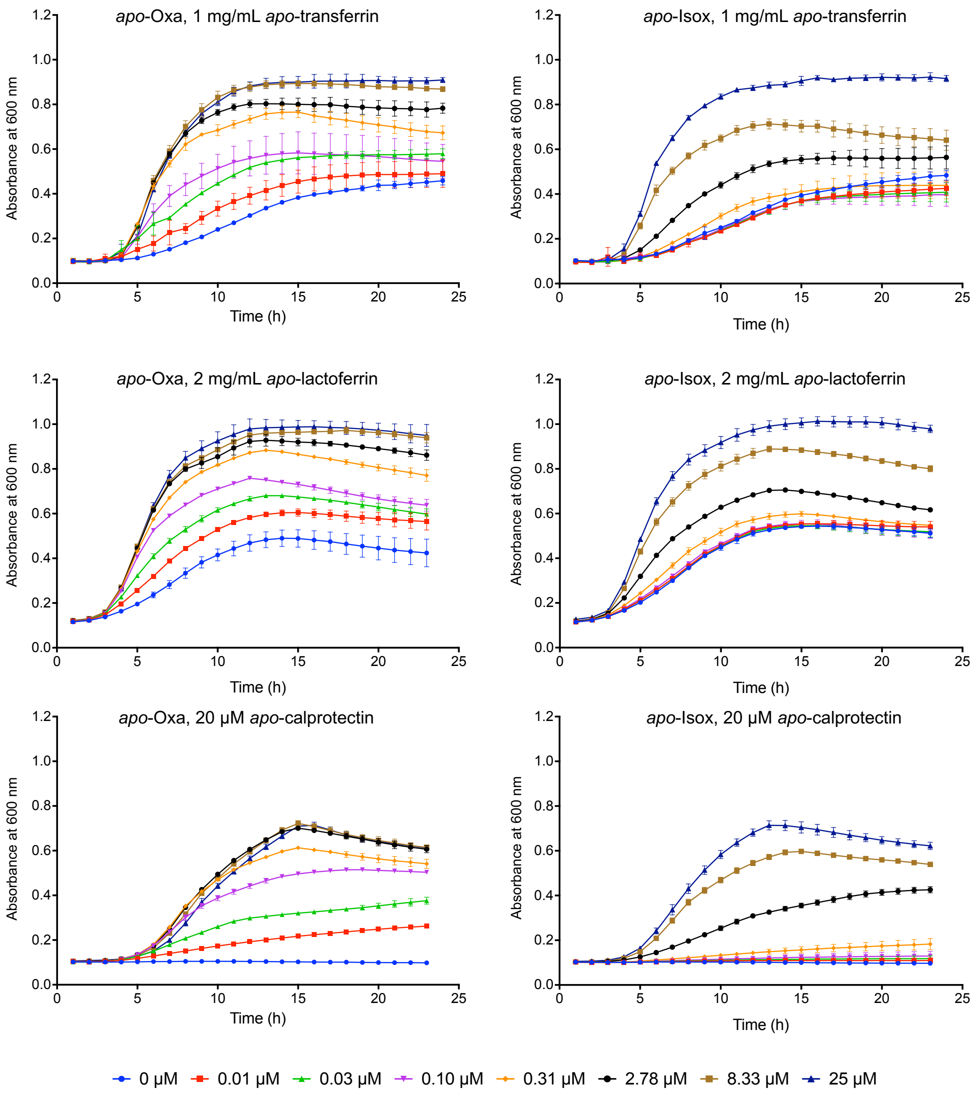

Fig S6. Growth curves of the *A. baumannii* Δ*basD* treated with either *apo*-Oxa or *apo*-Isox under iron-deficient conditions created by supplementation of the human metal sequestering proteins.

All experiments were conducted using the LB media containing 1 mg/mL *apo*-transferrin, 2 mg/mL *apo*-lactoferrin, or 20 μM *apo*-calprotectin. Error bars represent the standard deviations of independent triplicate experiments.
