# Supplementary material for: Distinctive Roles of Two Acinetobactin Isomers in Challenging Host Nutritional Immunity
Source: mBio. 2021 Sep 14;12(5):e02248-21. doi: 10.1128/mBio.02248-21 (PMC8546848; doi:10.1128/mBio.02248-21)
Supplement: FIG S8 [file mbio.02248-21-sf008.pdf]

```
17978     mnhnikglsmnnvsynrnfktgndqrinhrsfvlngvasaqmalrlgyalgtvfvlcasn 60
19606     mnqnikglsmnnvsynrnfktgndqrinhrsfvlngvasaqmalrlgyalgtvfvlcasn 60
AC12      mnqnikglsmnnvsynrnfktgndqrinhrsfvlngvasaqialrlgyalgtvfvlcasn 60
          **:**************************************:******************

17978     tyaavidnsaktleqqtaqtnvaalpaitvkaeqddtyaggqvssksssvgflgnktamet 120
19606     tyaavidnstktleqqtaqtnvaalpaitvkaeqddtyaggqvatssnvgflgskkfldt 120
AC12      tyaavidnsaktleqqtaqtnvaalpaitvkaeqddtyaggqvssksssvgflgnktvmet 120
          *********:*********************************::.*.*****.*. ::*

17978     pfntiaytekyiadqqakditdviaktdpsifstgaygviaesyairgfrsssvaagrdt 180
19606     pfntisytdkyiedkqakditeviaatdpsiytngasggwsenyyirgyasst----ndm 176
AC12      pfntiaytdtyiadkqakditsviaktdpsvftnaasggwsenyyirgfessp----sdm 176
          *****:**:.** *:******.*** ****:::..* *  :*.* ***: **      *

17978     smgglygiaplyraspemferidvlkgpsamlngmppgstlggtvnlvpkragddpltrf 240
19606     smnglfgitpfyrtspemfgrvevlkgpsallngmppagsvggtvnlvtkyaadepfarl 236
AC12      smnglfgitpyyrtspemfekinvlkgpsallngmpptgsiggtvdlttkratdepltrl 236
          **.**:**:* **:***** :::*******:****** .:****:*. * * *:*::*:

17978     tttymsdsqfgghldigrrfgenkefgvrfnsvyrdgegpikeqektthlfslgldwrgd 300
19606     tttymsdaqfgghvdvgrrfgenkefgvringmyrdgdaavndqskesrlfslgldwqge 296
AC12      tttymsdsqfgghldvgrrfgsdkefgvranavyrdgsgpvekqdlktelfslgmdwhgd 296
          *******:*****:*:*****.:****** *.:****.. ::.*.  :.*****:**:*:

17978     narlsadlytsrdhadginrgltilpgvnlpkapnpdtllnptwtvyntrdtggmirgey 360
19606     narvfvdaydaldhvdgvtrgvnvstavgipkppkadtllspdwgsvetkdkgamirgey 356
AC12      rarvstdlytskdrvdgvtrginlgkdiaipkppkpetllnpdwsfvdiqdkgamirgey 356
          .**: .* * : *:.**:.**:.:   : :** *: :***.* *   : :*.*.******

17978     dlndkwmayatagmskteyntlgaakteiqneagdikfniahlgfkyerksaevglrgkf 420
19606     dfsdqlmayaaygqstteykyngasagtitsstgtlsstlgqlafdvdkksadagfkgkf 416
AC12      dltnnvmgyatygqskteykyngamsatvldntgtfqtsigqltfnvdkksadvglkgkf 416
          *:.:: *.**: * *.***:  **    : ..:* :. .:.:* *. ::***:.*::***

17978     dtgavkhalalnathyretddeagirqgfpeg-drvtniyn--pnwgskpnrvpiapifs 477
19606     etgsvkhqwvanatyynhtqddygyriipgfsdpvitniydpnpnwgpkpeftpp-flfh 475
AC12      ntgavghqwvvnatyykhnqddygvrnvagae--wttnlyd--piwgkavpfnap-lisn 471
          :**:* *  . ***:*...:*: * *          **:*:  * **          :

17978     nkenltsvgladtmsvlqdqvqltlglryqnivteslngqgvrningrydknaitpaaai 537
19606     stlstssfgladtlsfaqdkvqltlglrhqtvkatss-----vntlpenaksattpgval 530
AC12      selqlnsygladtlsfiedrlqltlgvryqevessnlfv--ptstltkykknattpgaai 529
          .  . .* *****:*. :*::*****:*:* : : .        .    .  *.* **..*:

17978     lvkandhvslyanyieglssggtvptgqnyenegqifspfktkqmetgikidsgnftntf 597
19606     likatdkisvyanyiegltkgdqapat--asnpgeifppqktkqqelglkvdlgtfahtl 588
AC12      lfkatdkvsiyanyiegltkgdaaplt--aanyptifapyktkqtefgvkfdqgtfahtl 587
          *.**.*::*:********:.*. .*      *   ** * **** * *:*.* *.*::*:

17978     svfqiksrnsyvikstdekiksiygadgeqrnrgvewgfygspvdnirvmggltyidpki 657
19606     safeitkpssyldpsklvnnlptfvsdgeqrnrgiewsffgspiehvrlmggftyldpel 648
AC12      slfeikkpsaytdlvt-----niytsggeqrnrgiewsffgspienvrlmggasyiepev 642
          * *:*.. .:*    .        : :.*******:**.*:***:::*:*** :*::*::

17978     tktriaselgnvvagvpkvqaklgiegdvsyipg-ltltanataaskqyiyndnstsvpg 716
19606     tktksggndghtavavpknqaklgaewdtqvaqgtltlsgninavskqyinaentlsvpg 708
AC12      tktaiqsnegnmavgvpktqgklgvewdnevaqgvltlssnatavskqyidqentlhipg 702
          ***   .: *: ...*** *.*** * * .   * ***:.* .*.*****  :*:  :**

17978     revygigaryntkiaqfpvtirgnidnltnksywsmpqftnlmlgaprtyllsatidf   774
19606     rtlldvgarystkvedhpvtfraniynltnkaywaqpqltnlalgaprtymlsvsydf   766
AC12      rtlidvgarykttsisnhpitfkadinnlmnkaywgmpklsnlalgaprtymlsvsydf   760
          * : .:****.*.: :.*:*::.:* ** **:**. *:::** *******:**.: **
```

% identity	ATCC 17978	ATCC 19606	AC12
**ATCC 17978**	100.00	59.84	61.92
**ATCC 19606**	59.84	100.00	70.28
**AC12**	61.92	70.28	100.00

Fig S8. Sequence comparison among three *A. baumannii* BauA variants.

The sequences of three BauA variants from *A. baumannii* ATCC 17978, ATCC 19606, and AC12 were aligned using Clustal Omega, and the calculated identity matrix was shown as a table.
