# Supplementary material for: Distinctive Roles of Two Acinetobactin Isomers in Challenging Host Nutritional Immunity
Source: mBio. 2021 Sep 14;12(5):e02248-21. doi: 10.1128/mBio.02248-21 (PMC8546848; doi:10.1128/mBio.02248-21)
Supplement: TABLE S1 [file mbio.02248-21-st001.pdf]

Strain or plasmid	Relevant characteristic(s)[a]	Reference or source
Bacterial strains		
A. baumannii		
ATCC 19606^T	Type strain	
HKD01	ATCC 19606^T with Δ*basD*	Ref. (1)
HKD02	ATCC 19606^T with Δ*bauA*	Ref. (1)
OH747	ATCC 19606^T with Δ*bauB*	This study
OH764	ATCC 19606^T with Δ*bauD*	This study
OH766	ATCC 19606^T with Δ*bauF*	This study
OH1208	HKD01 with Δ*bauA*	This study
OH1040	$basD^+$ rescue in HKD01	This study
OH1046	$bauA^+$ rescue in HKD02	This study
OH1055	$bauB^+$ rescue in OH747	This study
OH1060	$bauD^+$ rescue in OH764	This study
E. coli		
DH5α λ *pir*	*supE44 △lacU169*(ϕ80 *lacZ*△M15) *hsdR17 recA1 endA1 gyrA96 thi-1 relA1 λpir* (phage lysogen); plasmid replication	Laboratory collection
S17-1 λ *pir*	*λpir* lysogen; *thi pro hsdR* $hsdM^+$ *recA* RP4-2 Tc::Mu-Km::Tn7; Tp^r Sm^r ; host for π-requiring plasmids; conjugal donor	Ref. (2)
Plasmid		
pUC4K	pUC4 with *nptI*; Ap^r, Km^r	Pharmacia
pHKD01	pDS132, multicloning sites; *ori*R6K *sacB*; Cm^r	Ref. (1)
pSE380	trc promoter expression vector; high copy number, IPTG inducible; Ap^r	Ref. (3)
pOH703	pHKD01 with Δ*bauB*::*nptI*; Cm^r, Km^r	This study
pOH710	pHKD01 with Δ*bauD*::*nptI*; Cm^r, Km^r	This study
pOH711	pHKD01 with Δ*bauF*::*nptI*; Cm^r, Km^r	This study
pOH1037	pHKD01 with *basD* coding region of *A. baumannii* ATCC19606 under control of its native promoter with *nptI*; Km^r	This study
pOH1041	pHKD01 with *bauA* coding region of *A. baumannii* ATCC19606 under control of its native promoter with *nptI*; Km^r	This study
pOH1051	pHKD01 with *bauB* coding region of *A. baumannii* ATCC19606 under control of its native promoter with *nptI*; Km^r	This study
pOH1053	pHKD01 with *bauD* coding region of *A. baumannii* ATCC19606 under control of its native promoter with *nptI*; Km^r	This study

[a]Tp^r, trimethoprim-resistant; Sm^r, streptomycin-resistant; Ap^r, ampicillin-resistant; Km^r, kanamycin-resistant; Cm^r, chloramphenicol-resistant.

References: (1) Appl Environ Microb (2015) 81:3357–3368. (2) Nat Biotechnol (1983) 1: 784–791. (3) Nucleic Acids Res (1991) 19: 4479–4484.

Table S1. Bacterial strains and plasmids used in this study.
