# Supplementary material for: Distinctive Roles of Two Acinetobactin Isomers in Challenging Host Nutritional Immunity
Source: mBio. 2021 Sep 14;12(5):e02248-21. doi: 10.1128/mBio.02248-21 (PMC8546848; doi:10.1128/mBio.02248-21)
Supplement: TABLE S2 [file mbio.02248-21-st002.pdf]

Primers[a]	Oligonucleotide sequence (5′→3′)[b,c]	Use
Gene deletion in *A. baumannii* ATCC 19606^{T}		
BauB01F	AGTTTCTATTTAGCCCTGATCGC	Amplification of upstream region of *bauB*
BauB01R	TTAACTAAGTGATGTGCCCAAAAATATGTATTCAAGCTCCAGATGAGTTG	
BauB02F	ATTTTTGGGCACATCACTTAGTTAA	Amplification of downstream region of *bauB*
BauB02R	GCAACACCTTCTTCACGAGGCAGACGCCATCAACATGATCTAAGGCA	
BauD01F	TTTCTGTCAGCACCAATGGC	Amplification of upstream region of *bauD*
BauD01R	TCATCCATAGCTTTGTAATACGCATCAAATCGCATCAGTGGCG	
BauD02F	ATGCGTATTACAAAGCTATGGATG	Amplification of downstream region of *bauD*
BauD02R	GCAACACCTTCTTCACGAGGCAGACCATTTGGACCAATGAGTGACG	
BauF01F	ATATATAACCCAAGCTTAATGATTACTATAGAG	Amplification of upstream region of *bauF*
BauF01R	GCAACACCTTCTTCACGAGGCAGACCCGACATATCATGATGAAACGG	
BauF02F	AAGTGCATTCATGGCACCAC	Amplification of downstream region of *bauF*
BauF02R	TAATCATTAAGCTTGGGTTATATATTTAAGTTTCTTTTACATACAAA-TAGGCAC	
Single copy complementation in *A. baumannii* mutants		
C-BauDCEBAF	GGCCTTTTTGCGTTTCTACAAACTCAAATGAATCCTGGTTTATCAAATGG	Amplification of $P_{bauDCEBA}$
C-BauDCEBAR	CAGTGGCGTGCCCCACT	
C-BauAF	CAGTGGCGTGCCCCACTTGAGCG-TAATGAATCAAAACATTAAAGGTCTTTCA	Amplification of *bauA* ORF
C-BauAR	TGCCGCCAGGCAAATTCTGTTTTATAATATGAATATCATAAAAAACCCGC	
C-BauBF	CAGTGGCGTGCCCCACTTGAGCGTAATGAACTGGAAGAAAAAGTATGGG	Amplification of *bauB* ORF
C-BauBR	TGCCGCCAGGCAAATTCTGTTTTATTTTTTATTAAC-TATCTGCTTAAAATTCATAGC	
C-BauDF	GGCCTTTTTGCGTTTCTACAAACTCAAATGAATCCTGGTTTATCAAATGG	Amplification of *bauD* ORF with its native pro-moter
C-BauDR	TGCCGCCAGGCAAATTCTGTTTTATTTATCTGAACTCCCTCAAAATGAGG	
C-BasDF	GGCCTTTTTGCGTTTC-TACAAACTCGTTTATACTTTATCTGTTTCCAAAATGATG	Amplification of *basD* ORF with its native promot-er
C-BasDR	TGCCGCCAGGCAAATTCTGTTTTATGACATTCTAAA-TATTCAATTTAATTTAATGGT	
Amplification of kanamycin-resistance cassette		
U1	GTCTGCCTCGTGAAGAAGGTG	Amplification of *nptI*
U2	GATCCGTCGACCTGCAGG	
Amplification of *rrmB* terminator		
R1	TCATCTGAGCAATGAGCAACCTTCCATAAAACAGAATTTGCCTGGCG	Amplification of *rrnB* terminator
R2	ACGACGACGCCGTGTGTTTGTTATCGAGTTTGTAGAAACGCAAAAAGGC	
Amplification of intergenic regions		
Int01F	CCTTGATAGGACACCAGTAGAAAGAC	Amplification of intergenic region I located be-tween DJ41_RS05115 and DJ41RS05120
Int01R	GGAAGGTTGCTCATTGCTCAG	
Int02F	GATAACAAACACACGGCGTCG	Amplification of intergenic region II located be-tween DJ41_RS05115 and DJ41RS05120
Int02R	GCAACACCTTCTTCACGAGGCAGACTCCTCATCGCGTAGATGATTTAG	

[a]The primers were designed using the genomic sequences of *A. baumannii* ATCC 19606 (GenBank accession number ACQB00000000).

[b]Regions of oligonucleotides that are not complementary to the corresponding templates are underlined.

[c]Regions of oligonucleotides that are subjected to restriction enzymes are underlined and italicized.

Table S2. Oligonucleotides used in this study.
